# Identifying the Severity of Heart Valve Stenosis and Regurgitation Among a Diverse Population Within an Integrated Health Care System: Natural Language Processing Approach

**DOI:** 10.2196/60503

**Published:** 2024-09-30

**Authors:** Fagen Xie, Ming-sum Lee, Salam Allahwerdy, Darios Getahun, Benjamin Wessler, Wansu Chen

**Affiliations:** 1 Department of Research and Evaluation Kaiser Permanente Southern California Pasadena, CA United States; 2 Department of Cardiology Los Angeles Medical Center Kaiser Permanente Southern California Pasadena, CA United States; 3 Department of Clinical Science Kaiser Permanente Bernard J Tyson School of Medicine Pasadena, CA United States; 4 Division of Cardiology Tufts Medical Center Boston, MA United States

**Keywords:** echocardiography report, heart valve, stenosis, regurgitation, natural language processing, algorithm

## Abstract

**Background:**

Valvular heart disease (VHD) is a leading cause of cardiovascular morbidity and mortality that poses a substantial health care and economic burden on health care systems. Administrative diagnostic codes for ascertaining VHD diagnosis are incomplete.

**Objective:**

This study aimed to develop a natural language processing (NLP) algorithm to identify patients with aortic, mitral, tricuspid, and pulmonic valve stenosis and regurgitation from transthoracic echocardiography (TTE) reports within a large integrated health care system.

**Methods:**

We used reports from echocardiograms performed in the Kaiser Permanente Southern California (KPSC) health care system between January 1, 2011, and December 31, 2022. Related terms/phrases of aortic, mitral, tricuspid, and pulmonic stenosis and regurgitation and their severities were compiled from the literature and enriched with input from clinicians. An NLP algorithm was iteratively developed and fine-trained via multiple rounds of chart review, followed by adjudication. The developed algorithm was applied to 200 annotated echocardiography reports to assess its performance and then the study echocardiography reports.

**Results:**

A total of 1,225,270 TTE reports were extracted from KPSC electronic health records during the study period. In these reports, valve lesions identified included 111,300 (9.08%) aortic stenosis, 20,246 (1.65%) mitral stenosis, 397 (0.03%) tricuspid stenosis, 2585 (0.21%) pulmonic stenosis, 345,115 (28.17%) aortic regurgitation, 802,103 (65.46%) mitral regurgitation, 903,965 (73.78%) tricuspid regurgitation, and 286,903 (23.42%) pulmonic regurgitation. Among the valves, 50,507 (4.12%), 22,656 (1.85%), 1685 (0.14%), and 1767 (0.14%) were identified as prosthetic aortic valves, mitral valves, tricuspid valves, and pulmonic valves, respectively. Mild and moderate were the most common severity levels of heart valve stenosis, while trace and mild were the most common severity levels of regurgitation. Males had a higher frequency of aortic stenosis and all 4 valvular regurgitations, while females had more mitral, tricuspid, and pulmonic stenosis. Non-Hispanic Whites had the highest frequency of all 4 valvular stenosis and regurgitations. The distribution of valvular stenosis and regurgitation severity was similar across race/ethnicity groups. Frequencies of aortic stenosis, mitral stenosis, and regurgitation of all 4 heart valves increased with age. In TTE reports with stenosis detected, younger patients were more likely to have mild aortic stenosis, while older patients were more likely to have severe aortic stenosis. However, mitral stenosis was opposite (milder in older patients and more severe in younger patients). In TTE reports with regurgitation detected, younger patients had a higher frequency of severe/very severe aortic regurgitation. In comparison, older patients had higher frequencies of mild aortic regurgitation and severe mitral/tricuspid regurgitation. Validation of the NLP algorithm against the 200 annotated TTE reports showed excellent precision, recall, and F1-scores.

**Conclusions:**

The proposed computerized algorithm could effectively identify heart valve stenosis and regurgitation, as well as the severity of valvular involvement, with significant implications for pharmacoepidemiological studies and outcomes research.

## Introduction

Valvular heart disease (VHD) is a leading cause of cardiovascular morbidity and mortality worldwide [1-3] and poses a substantial health care and economic burden on health care systems [4,5]. The prevalence of VHD, especially aortic stenosis, is expected to rapidly increase in the United States and Europe due to population aging [4,5]. Accurate assessments of the burden of VHD are increasingly relevant as the treatment options for these patients continue to expand. VHD research based on administrative diagnostic codes shows incomplete identification [6] or inaccuracy of coding [7]. Accurate and complete identification of VHD based on information from echocardiography reports other than diagnosis codes has the potential to facilitate patient care and VHD-related cardiovascular research.

Advances in diagnostic imaging technologies have greatly improved the precision and efficiency of assessing heart valve disorders [8,9]. Echocardiography is the primary imaging modality for evaluating valve structure and function and assessing the severity and hemodynamic consequences of VHDs. Transthoracic echocardiography (TTE) provides key insights into the mechanisms of VHDs [8]. The wealth of data and information generated by the interpretations of echocardiographic studies significantly aids clinical management and research. Although the format of echocardiography reports is often templated, the content in each section remains as free text. This presents a challenge for systematic analysis, necessitating advanced natural language processing (NLP) techniques to transform from unstructured into structured and analyzable data [10].

Over the past years, applications of NLP algorithms or systems have been developed to automatically extract clinical information from free-text clinical notes [11-13]. Rule-based or machine learning–based NLP studies [6,14-22] have attempted to extract information about valve severity and related measurements from echocardiography reports. Most of these studies have concentrated on extracting some specific conditions and measurements, such as aortic stenosis and peak velocity. Two exceptions are Nath et al [18], who created EchoInfer, a system capable of extracting a set of data elements (~80) reported in echocardiography reports, and Dong et al [19], who developed an NLP system that extracts ~43 data elements described in echocardiography reports. Although both systems extracted elements relevant to VHD, the performance was based on the overall data elements rather than the clinically relevant feature of the severity of individual VHD. Additionally, the small training and validation samples in both studies limited the capabilities to accurately assess performance for less common VHDs, such as mitral valve, tricuspid valve, and pulmonic valve stenosis. The purpose of this study was twofold: (1) to develop and validate a computerized algorithm for extracting the severity of stenosis and regurgitation of the 4 heart valves (aortic valve, mitral valve, tricuspid valve, and pulmonic valve) and (2) to apply the validated algorithm to all TTE reports within the large integrated Kaiser Permanente Southern California (KPSC) health care system to estimate the frequencies of VHD across a diverse population.

## Methods

### Study Setting and Population

The study subjects were health plan enrollees of the KPSC, an integrated health care system providing comprehensive medical services to 4.8 million members across 15 large medical centers and more than 250 medical offices throughout Southern California. The demographic characteristics of KPSC members are diverse and largely representative of the residents in Southern California [23], with health insurance through group plans, individual plans, Medicare, and Medicaid. Patients aged 18 years or older who underwent at least 1 TTE within the KPSC system between January 1, 2011, and December 31, 2022, were included in this study.

### Ethical Considerations

The KPSC Institutional Review Board reviewed and approved the study protocol, with a waiver of the requirement for informed consent (approval number 13490). The study complied with the Health Insurance Portability and Accountability Act. Only authorized persons were provided access permission to perform all analyses.

### NLP Algorithm and Process

**Figure 1:**
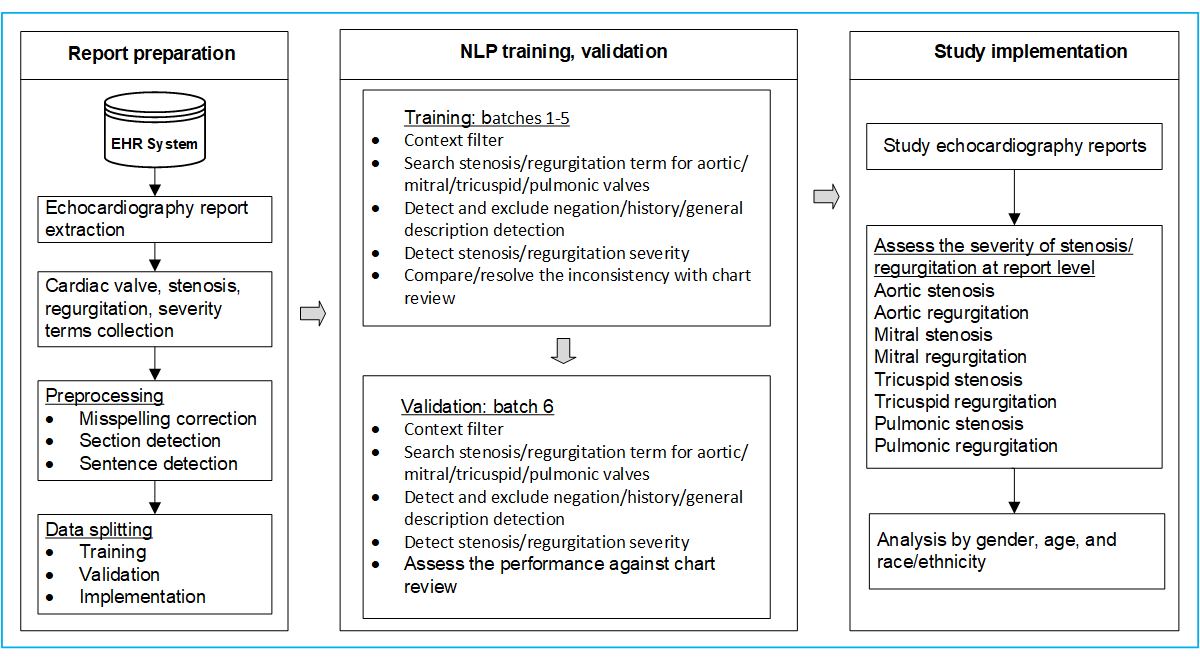
Schematic processing diagram describing the NLP algorithm for identifying heart valve stenosis and regurgitation from TTE reports. EHR: electronic health record; NLP: natural language processing; TTE: transthoracic echocardiography.

Figure 1 outlines the steps for identifying valvular stenosis and regurgitation, and detailed descriptions follow later.

#### Echocardiography Report Extraction and Annotation

The TTE reports during the study period were extracted from the KPSC’s electronic health record (EHR) system. These reports were written by physicians and were generally structured in a templated format. Most reports contain the following sections: (1) title, patient demographics, procedure performed, performing provider, and procedure indication; (2) exam quality; (3) dimensions/measurements; (4) findings/results; (5) impression; (6) miscellaneous; (7) summary/conclusion; and (8) physician signature. Despite the templated structure, the content within each section is in free-text format, and the report can have a varying order of or incomplete sections. Examples of deidentified TTE reports are included in Table S1 in Multimedia Appendix 1.

An initial list of phrases and terms related to capturing stenosis, regurgitation, and severity of the 4 heart valves was compiled based on the input of the study cardiologist, published case definitions, and ontologies [6,18,19,24] and enriched by the training data set to capture additional linguistic variations, such as abbreviations and misspellings. The collected terms are listed in Table S2 in Multimedia Appendix 1.

To effectively capture the severity of the rare heart valve stenosis described in the TTE reports, 2 sets of TTE reports were prepared for annotation and algorithm training. The first data set contained a total of 800 TTE reports, of which 200 (25%) were randomly selected from each of the 4 aortic valve peak velocity groups (≤2.5, 2.6-2.9, 3.0-4.0, ≥4.0 m/s) instead of simple random selection from the extracted entire TTE reports (data set 1). The second data set contained another sample with 400 TTE reports based on diagnosis codes (data set 2): 134 (33.5%) reports randomly selected for patients with a mitral stenosis diagnosis (International Classification of Diseases 10th Revision [ICD-10] code I05.0 or I05.2), 133 (33.3%) reports randomly selected for patients with a tricuspid stenosis diagnosis (ICD-10 code I07.0, I07.2, I36.0, or I36.2), and 133 (33.3%) reports randomly selected for patients with a pulmonic stenosis diagnosis (ICD-10 code I37.0 or I37.2). Both data sets were manually reviewed by an experienced board-certified cardiologist and a medical student to record the presence/absence (Table S2 in Multimedia Appendix 1) and severity (Table S3 in Multimedia Appendix 1) of stenosis and regurgitation of the 4 heart valves. These annotated TTE reports were split into 6 batches, each containing 200 reports. The first batch was reviewed by both annotators to ensure quality and consistency. The rest were equally divided between the 2 annotators.

### NLP Algorithm Development

We first divided the reports in the annotated data sets into the sections of echocardiography report extraction and annotation described before and then subsectioned them based on titles and subtitles. Each subsection uniquely captured information about a specific valve (aortic, mitral, tricuspid, or pulmonic). The selected sections and subsections were then preprocessed through the letter lowercase conversion, misspelled word correction (as shown in Table S4 in Multimedia Appendix 1), and tokenization (ie, segmenting text into linguistic units, such as words and punctuations) [25] for further NLP processing. The study terms/phrases and their abbreviations and acronyms were collected by the cardiologist before NLP development. For each of the study terms/phrases, misspelled word correction was performed by manually examining the top 100 similar words derived from a trained deep learning word2vec model [26,27] based on the study corpus; 100% of data set 1 and 50% of data set 2 were used for training, and 50% of data set 2 was used for validation.

We used the annotated reports to develop a rule-based computerized algorithm via an iterative process to determine the presence/absence and severity status of stenosis and regurgitation in the 4 heart valves (aortic, mitral, tricuspid, and pulmonic). Table S5 in Multimedia Appendix 1 summarizes the included sections and subsections using which the following search steps were applied. The process was applied to each sentence within the included sections or subsections:

- Search for terms associated with stenosis and regurgitation. The status was labeled as “no evidence” if no relevant term was found (Table S1 in Multimedia Appendix 1).
- If a relevant term is found, search for the negated terms associated with the identified stenosis and regurgitation terms. If a negation was found (eg, no aortic stenosis, without evidence of aortic stenosis), the identified stenosis or regurgitation term was ignored.
- Search for history terms (eg, a prior study showed trace mitral regurgitation) associated with the identified stenosis and regurgitation terms. If an associated history term was detected, the detected stenosis and regurgitation term was also ignored (Table S6 in Multimedia Appendix 1).
- Search for severity terms. If no severity term was found, the sentence was labeled “unknown severity.” If multiple severity terms were detected, the severity of the report was assigned based on the following priority: prosthetic, very severe, severe, moderate to severe, moderate, mild to moderate, mild, trace to mild, trace, and sclerosis. Trace to mild and trace were only applied for regurgitation, while sclerosis was only applied for aortic stenosis (Table S2 in Multimedia Appendix 1).

Discordant cases between the computerized algorithm and manually annotated labels were reviewed and adjudicated by the cardiologist. If the adjudicated results differed from the computerized results within each round, they were used to refine the algorithm and process.

### NLP Algorithm Validation

The results from the final computerized algorithm were compared with the manually annotated results in the validation data set. The proportions of true-positive (TP), false-positive (FP), and false-negative (FN) cases were used to estimate sensitivity, the positive predicted value (PPV), and the overall *F*_1_-score (a measure of the overall model fit). Sensitivity was defined as the proportion of reports correctly labeled by the computerized algorithm (TP) among all reports (TP+FN) ascertained by chart review. The PPV was defined as the proportion of reports correctly labeled (TP) among all those labeled by the computerized algorithm (TP+FP). The overall accuracy of the *F*_1_-score for each comparison was calculated via the standard formula 2 × PPV × sensitivity/(PPV + sensitivity).

### Estimating the Severity of Stenosis and Regurgitation at the Report Level

The finalized computerized algorithm was implemented via Python 3.10 to process the entire study set of TTE reports. The status and severity level of stenosis and regurgitation for each of the 4 heart valves (aortic, mitral, tricuspid, and pulmonic) were reported for all TTE reports during the study period. In TTE reports with VHDs detected, the severity levels of the diseases at the report level were summarized by age group (18-49, 50-64, 65-79, and ≥80 years), sex, and race/ethnicity (non-Hispanic White, non-Hispanic Black, non-Hispanic Asian/Pacific Islander, non-Hispanic Native American, Hispanic, multiple races, other/unknown).

## Results

### Performance Assessment of the NLP Algorithm

The performance of the computerized algorithm against the manually annotated results based on the validation data set is summarized in Table 1 for stenosis and Table 2 for regurgitation. The PPV, sensitivity, and *F*_1_-score of having positive stenosis and regurgitation were 100%, 100%, and 1 for aortic, mitral, and tricuspid valves; 96.2%, 96.2%, and 0.96 for pulmonic stenosis, respectively; and 97.0%, 98.5%, and 0.98 for pulmonic regurgitation, respectively. The PPV, sensitivity, and *F*_1_-score of prosthetic valves were also 100%, 100%, and 1 for aortic, mitral, and tricuspid valves and 92.3%, 92.3%, and 0.92 for pulmonic valves, respectively. For TTE reports with specific severity detected, the PPV was 100% for most of the severe categories, with several exceptions (eg, 80% for severe mitral stenosis and 50% for unknown severity pulmonic stenosis; Table 1). Sensitivity was also 100% for most of the severe categories, with several exceptions (eg, 87.5% for moderate-to-severe mitral stenosis; Table 1).

**Table 1:** Computerized algorithm performance for stenosis against adjudicated chart review results for the 200 TTE^a^ reports in the validation data set.

Valve and severity status		TP^b^	FP^c^	FN^d^	PPV^e^ (%)	Sensitivity (%)	*F*_1_-score
**Aortic valve**							
	No/no evidence	113	0	1	100.0	99.1	1.00
	Prosthetic	28	0	0	100.0	100.0	1.00
	Sclerosis	28	1	0	96.6	100.0	0.98
**Aortic valve severity detected**		31	0	0	100.0	100.0	1.00
	Mild	15	0	0	100.0	100.0	1.00
	Mild to moderate	1	0	0	100.0	100.0	1.00
	Moderate	8	0	0	100.0	100.0	1.00
	Moderate to severe	0	0	0	—^f^	—	—
	Severe	7	0	0	100.0	100.0	1.00
	Very severe	0	0	0	—	—	—
	Unknown severity	0	0	0	—	—	—
**Mitral valve**							
	No/no evidence	135	0	0	100.0	100.0	1.00
	Prosthetic	17	0	0	100.0	100.0	1.00
**Mitral valve severity detected**		48	0	0	100.0	100.0	1.00
	Mild	7	0	0	100.0	100.0	1.00
	Mild to moderate	7	0	0	100.0	100.0	1.00
	Moderate	21	0	0	100.0	100.0	1.00
	Moderate to severe	7	0	1	100.0	87.5	0.93
	Severe	4	1	0	80.0	100.0	0.89
	Very severe	0	0	0	—	—	—
	Unknown severity	1	0	0	100.0	100.0	1.00
**Tricuspid valve**							
	No/no evidence	185	0	0	100.0	100.0	1.00
	Prosthetic	10	0	0	100.0	100.0	1.00
**Tricuspid valve severity detected**		5	0	0	100.0	100.0	1.00
	Mild	1	0	0	100.0	100.0	1.00
	Mild to moderate	0	0	0	—	—	—
	Moderate	1	0	0	100.0	100.0	1.00
	Moderate to severe	2	0	0	100.0	100.0	1.00
	Severe	0	0	0	—	—	—
	Very severe	0	0	0	—	—	—
	Unknown severity	1	0	0	100.0	100.0	1.00
**Pulmonic valve**							
	No/no evidence	159	2	2	98.8	98.8	0.99
	Prosthetic	12	1	1	92.3	92.3	0.92
**Pulmonic valve severity detected**		25	1	1	96.2	96.2	0.96
	Mild	18	0	1	100.0	94.7	0.97
	Mild to moderate	4	0	0	100.0	100.0	1.00
	Moderate	1	0	0	100.0	100.0	1.00
	Moderate to severe	0	0	0	—	—	—
	Severe	1	0	0	100.0	100.0	1.00
	Very severe	1	0	0	100.0	100.0	1.00
	Unknown severity	2	1	0	50.0	100.0	0.67

^a^TTE: transthoracic echocardiography.

^b^TP: true positive. Both the computerized algorithm and the chart review had the same result.

^c^FP: false positive. The computerized algorithm was identified as yes, but the chart review was labeled as no.

^d^FN: false negative. The chart review was labeled as yes, but the computerized algorithm was identified as no.

^e^PPV: positive predicted value.

^f^Not applicable.

**Table 2:** Computerized algorithm performance for regurgitation against adjudicated chart review results for the 200 TTE^a^ reports in the validation data set.

Valve and severity status		TP^b^	FP^c^	FN^d^	PPV^e^ (%)	Sensitivity (%)	*F*_1_-score
**Aortic valve**							
	No/no evidence	109	0	0	100.0	100.0	1.00
	Prosthetic	28	0	0	100.0	100.0	1.00
	Sclerosis	0	0	0	—^f^	—	—
**Aortic valve severity detected**		63	0	0	100.0	100.0	1.00
	Trace	26	0	0	100.0	100.0	1.00
	Trace to mild	0	0	0	—	—	—
	Mild	23	0	0	100.0	100.0	1.00
	Mild to moderate	7	0	0	100.0	100.0	1.00
	Moderate	5	0	0	100.0	100.0	1.00
	Moderate to severe	1	0	0	100.0	100.0	1.00
	Severe	1	0	0	100.0	100.0	1.00
	Very severe	0	0	0	—	—	—
	Unknown severity	0	0	0	—	—	—
**Mitral valve**							
	No/no evidence	60	0	0	100.0	100.0	1.00
	Prosthetic	17	0	0	100.0	100.0	1.00
**Mitral valve severity detected**		123	0	0	100.0	100.0	1.00
	Trace	47	0	0	100.0	100.0	1.00
	Trace to mild	2	0	1	100.0	66.7	0.80
	Mild	35	1	0	97.2	100.0	0.99
	Mild to moderate	15	0	0	100.0	100.0	1.00
	Moderate	13	0	1	100.0	92.9	0.96
	Moderate to severe	3	0	0	100.0	100.0	1.00
	Severe	5	1	0	83.3	100.0	0.91
	Very severe	0	0	0	—	—	—
	Unknown severity	0	0	0	—	—	—
**Tricuspid valve**							
	No/no evidence	41	0	0	100.0	100.0	1.00
	Prosthetic	10	0	0	100.0	100.0	1.00
**Tricuspid valve severity detected**		149	0	0	100.0	100.0	1.00
	Trace	46	0	1	100.0	97.9	0.99
	Trace to mild	2	1	0	66.7	100.0	0.80
	Mild	43	0	0	100.0	100.0	1.00
	Mild to moderate	22	0	0	100.0	100.0	1.00
	Moderate	19	0	0	100.0	100.0	1.00
	Moderate to severe	5	0	1	100.0	83.3	0.91
	Severe	10	1	0	90.9	100.0	0.95
	Very severe	0	0	0	—	—	—
	Unknown severity	0	0	0	—	—	—
**Pulmonic valve**							
	No/no evidence	121	0	1	100.0	99.2	1.00
	Prosthetic	12	1	1	92.3	92.3	0.92
**Pulmonic valve severity detected**		64	2	1	97.0	98.5	0.98
	Trace	24	0	1	100.0	96.0	0.98
	Trace to mild	0	0	0	—	—	—
	Mild	28	1	0	96.6	100.0	0.98
	Mild to moderate	3	0	0	100.0	100.0	1.00
	Moderate	6	0	0	100.0	100.0	1.00
	Moderate to severe	1	0	0	100.0	100.0	1.00
	Severe	2	1	0	66.7	100.0	0.80
	Very severe	0	0	0	—	—	—
	Unknown severity	0	0	0	—	—	—

^a^TTE: transthoracic echocardiography.

^b^TP: true positive. Both the computerized algorithm and the chart review had the same result.

^c^FP: false positive. The computerized algorithm was identified as yes, but the chart review was labeled as no.

^d^FN: false negative. The chart review was labeled as yes, but the computerized algorithm was identified as no.

^e^PPV: positive predicted value.

^f^Not applicable.

### Estimating the Severity of Stenosis and Regurgitation at the Report Level

**Figure 2:**
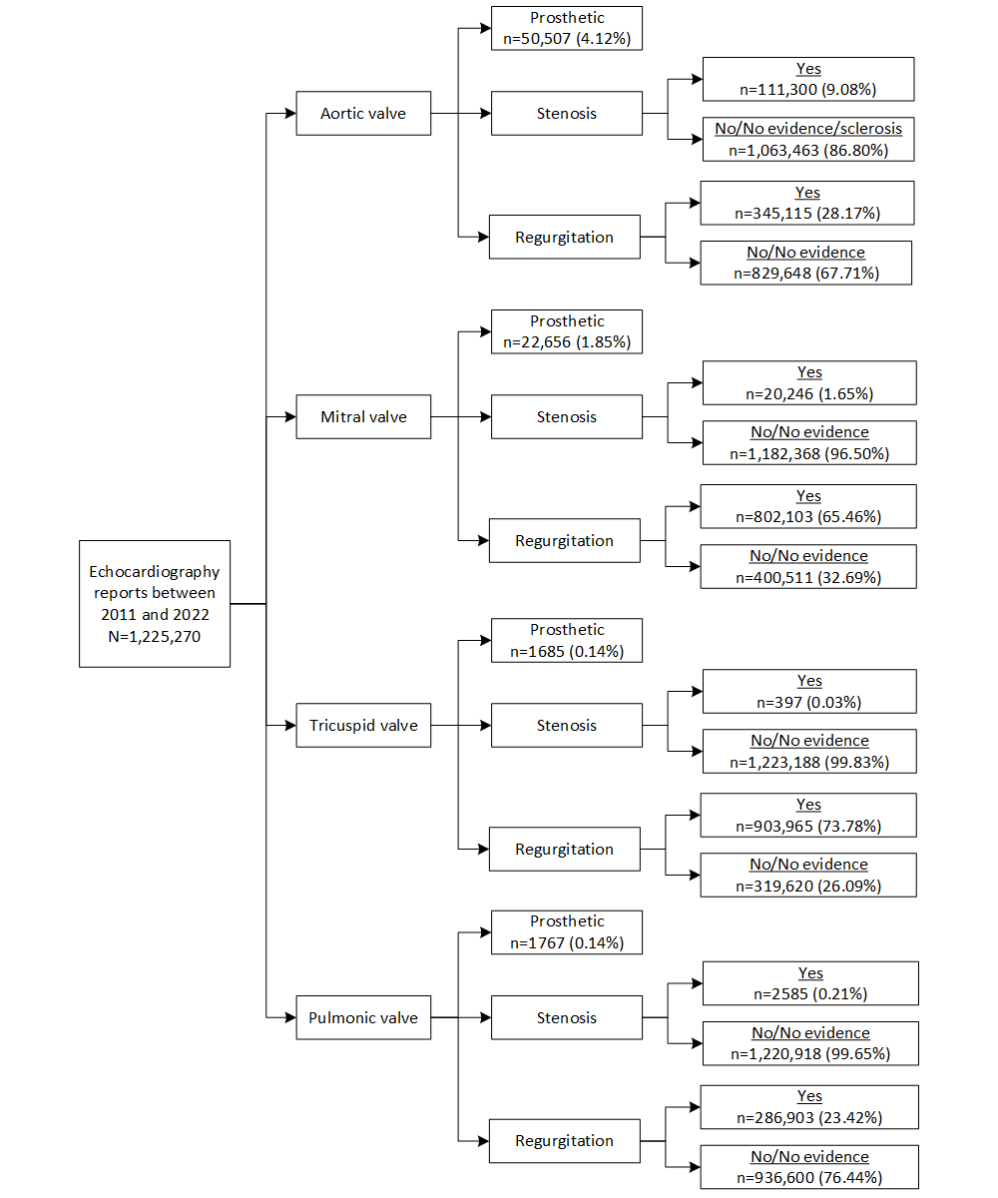
The NLP algorithm identified frequencies of stenosis and regurgitation by heart valve based on TTE reports in the KPSC setting during 2011-2022. KPSC: Kaiser Permanente Southern California; NLP: natural language processing; TTE: transthoracic echocardiography.

**Figure 3:**
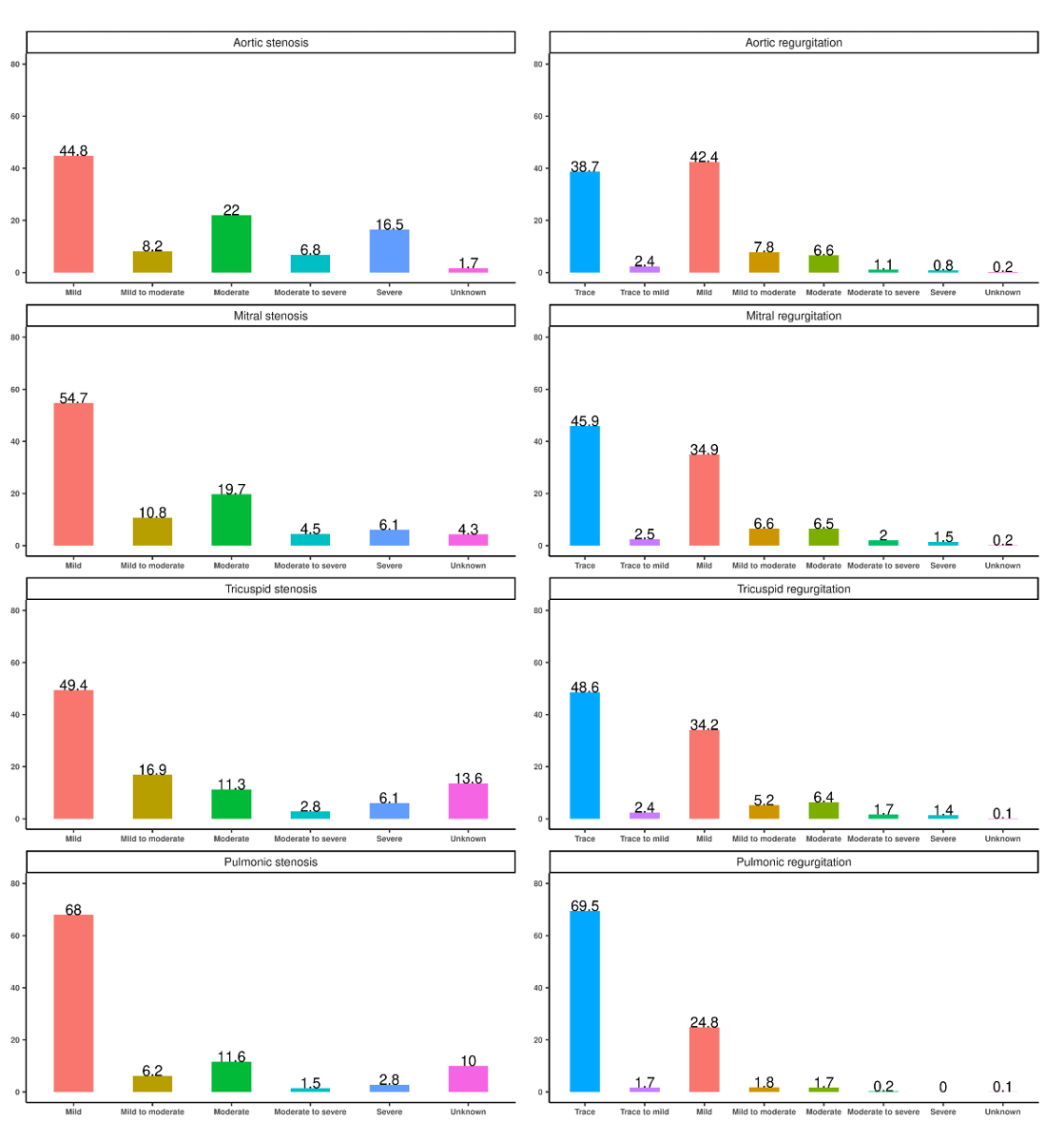
Percentage distribution of the severity of stenosis and regurgitation by heart valve based on TTE reports in the KPSC setting during 2011-2022. KPSC: Kaiser Permanente Southern California; TTE: transthoracic echocardiography. A higher resolution version of this image is available in Multimedia Appendix 2.

A total of 1,225,270 TTE reports among 677,106 patients were extracted from the KPSC EHR system during the study period. Slightly more than half (n=621,237, 50.7%, data not shown) of them were for male patients. The median age at the time of the echocardiogram was 67 years (IQR 55-77). The mean number of TTEs performed per patient was 1.8 (SD 1.6) during the study period (data not shown). The distributions of the stenosis and regurgitation severity across the TTE reports identified by the NLP algorithm and process are summarized in Figure 2. Of the 1,225,270 TTE reports, 111,300 (9.08%), 20,246 (1.65%), 397 (0.03%), 2585 (0.21%), 345,115 (28.17%), 802,103 (65.46%), 903,965 (73.78%), and 286,903 (23.42%) reports had evidence of aortic stenosis, mitral stenosis, tricuspid stenosis, pulmonic stenosis, aortic regurgitation, mitral regurgitation, tricuspid regurgitation, and pulmonic regurgitation, respectively. In addition, 50,507 (4.12%), 22,656 (1.85%), 1685 (0.14%), and 1767 (0.14%) of the heart valves were identified as prosthetic aortic, mitral, tricuspid, and pulmonic valves, respectively. The distribution of severity levels among each identified VHD is shown in Figure 3. Mild and moderate were the most common severity levels of heart valve stenosis, while trace and mild were the most common ones for regurgitation. More details can be found in Table S7 in Multimedia Appendix 1.

In TTE reports with VHDs detected, the severity level of the diseases stratified by sex, race/ethnicity, and age group at the time of the TTE are presented in Tables 3-6 for stenosis and Tables 7-10 for regurgitation. Males had a higher frequency of aortic stenosis and all 4 valvular regurgitations, while females had more mitral, tricuspid, and pulmonic stenosis. Non-Hispanic Whites had the highest frequency of all 4 valvular stenosis and regurgitations. The distribution of stenosis and regurgitation severity was similar across race/ethnicity groups. The frequencies of aortic and mitral stenosis increased with age, whereas the frequencies of tricuspid and pulmonic stenosis decreased with age. The frequency of valvular regurgitation increased with age for all 4 heart valves. Among the TTE reports with stenosis detected, younger patients were more likely to have mild aortic stenosis, while older patients were more likely to have severe aortic stenosis. However, the frequencies of mitral stenosis were opposite (milder mitral stenosis in older patients and more severe mitral stenosis in younger patients). In contrast, for TTE reports with regurgitation detected, younger patients had a higher frequency of severe/very severe aortic regurgitation, while older patients had higher frequencies of mild aortic regurgitation and severe/very severe mitral/tricuspid regurgitation. The distribution of severity can be found in Table S8 in Multimedia Appendix 1.

**Table 3:** Severity of aortic stenosis captured in the 1,225,270 TTE^a^ reports in the KPSC^b^ health care system during 2011-2022 by VHD^c^, sex, race/ethnicity, and age at TTE time.

Characteristics			Severity of detected VHD								
			Mild/mild to moderate, n (%)		Moderate/moderate to severe, n (%)		Severe/very severe, n (%)		Unknown severity, n (%)		Total, N
**Sex**											
	Female	27,872 (54.8)		13,967 (27.5)		8132 (16.2)		887 (1.7)		50,858	
	Male	31,098 (51.5)		18,163 (30.1)		10,168 (16.8)		1021 (1.7)		60,440	
**Age group (years)**											
	18-49	1797 (58.3)		838 (27.2)		302 (9.8)		147 (4.8)		3084	
	50-64	7140 (55.3)		3367 (26.1)		2098 (16.3)		290 (2.3)		12,895	
	65-79	27,855 (55.1)		14,255 (28.2)		8142 (18.1)		803 (1.6)		50,548	
	≥80	22,178 (49.5)		13,672 (30.5)		8255 (18.5)		668 (1.5)		44,773	
**Race/ethnicity**											
	Non-Hispanic White	34,039 (51.5)		19,612 (29.7)		11,443 (17.3)		1019 (1.5)		66,113	
	Non-Hispanic Black	4917 (56.2)		2473 (28.3)		1197 (13.7)		167 (1.9)		8754	
	Hispanic	14,008 (53.1)		7452 (28.2)		4376 (16.6)		533 (2.0)		26,368	
	Non-Hispanic Asian/Pacific Islander	5323 (60.7)		2228 (25.4)		1065 (12.2)		167 (1.9)		8783	
	Non-Hispanic Native American	116 (53.0)		52 (23.8)		46 (21.0)		5 (2.3)		219	
	Multiple	71 (50.8)		41 (29.2)		21 (15.0)		7 (5.0)		140	
	Other/unknown	496 (53.7)		274 (29.7)		143 (15.5)		10 (1.1)		923	

^a^TTE: transthoracic echocardiography.

^b^KPSC: Kaiser Permanente Southern California.

^c^VHD: valvular heart disease.

**Table 4:** Severity of mitral stenosis captured in the 1,225,270 TTE^a^ reports in the KPSC^b^ health care system during 2011-2022 by VHD^c^, sex, race/ethnicity, and age at TTE time.

Characteristics		Severity of detected VHD				
		Mild/mild to moderate, n (%)	Moderate/moderate to severe, n (%)	Severe/very severe, n (%)	Unknown severity, n (%)	Total, N
**Sex**						
	Female	9098 (64.4)	3525 (25.0)	929 (6.5)	579 (4.1)	14,130
	Male	4163 (68.1)	1371 (22.4)	298 (4.9)	284 (4.6)	6116
**Age group (years)**						
	18-49	514 (52.9)	275 (28.3)	105 (11.0)	75 (7.7)	971
	50-64	1506 (55.2)	807 (29.6)	279 (9.9)	145 (5.3)	2728
	65-79	5672 (65.8)	2077 (24.0)	527 (6.1)	356 (4.1)	8632
	≥80	5569 (70.4)	1737 (22.0)	323 (4.0)	286 (3.6)	7915
**Race/ethnicity**						
	Non-Hispanic White	7059 (68.8)	2360 (23.1)	467 (4.5)	375 (3.7)	10,261
	Non-Hispanic Black	1139 (66.0)	382 (22.0)	124 (7.2)	84 (4.9)	1725
	Hispanic	3652 (63.0)	1440 (24.8)	416 (7.2)	293 (5.1)	5801
	Non-Hispanic Asian/Pacific Islander	1286 (56.7)	676 (29.8)	204 (9.0)	102 (4.5)	2268
	Non-Hispanic Native American	20 (57.1)	9 (25.7)	4 (11.4)	2 (5.7)	35
	Multiple	21 (75.0)	6 (21.4)	0	1 (3.6)	28
	Other/unknown	85 (66.4)	26 (20.3)	12 (9.4)	5 (3.9)	128

^a^TTE: transthoracic echocardiography.

^b^KPSC: Kaiser Permanente Southern California.

^c^VHD: valvular heart disease.

**Table 5:** Severity of tricuspid stenosis captured in the 1,225,270 TTE^a^ reports in the KPSC^b^ health care system during 2011-2022 by VHD^c^, sex, race/ethnicity, and age at TTE time.

Characteristics		Severity of detected VHD				
		Mild/mild to moderate, n (%)	Moderate/moderate to severe, n (%)	Severe/very severe, n (%)	Unknown severity, n (%)	Total, N
**Sex**						
	Female	156 (69.3)	28 (12.5)	11 (4.9)	30 (13.3)	225
	Male	107 (62.3)	28 (16.3)	13 (7.6)	24 (14.0)	172
**Age group (years)**						
	18-49	112 (85.5)	5 (3.9)	4 (3.1)	10 (7.6)	131
	50-64	40 (56.3)	12 (16.9)	11 (15.5)	8 (11.3)	71
	65-79	75 (59.7)	25 (20.1)	5 (4.0)	20 (16.1)	124
	≥80	37 (52.2)	14 (19.7)	4 (5.6)	16 (22.5)	71
**Race/ethnicity**						
	Non-Hispanic White	116 (68.6)	23 (13.7)	9 (5.3)	21 (12.4)	169
	Non-Hispanic Black	38 (77.6)	4 (8.2)	4 (8.2)	3 (6.1)	49
	Hispanic	87 (64.0)	24 (17.6)	8 (5.9)	17 (12.5)	136
	Non-Hispanic Asian/Pacific Islander	19 (50.0)	4 (10.5)	3 (7.9)	12 (31.6)	38
	Non-Hispanic Native American	0	0	0	1 (100.0)	1
	Multiple	0	0	0	0	0
	Other/unknown	3 (75.0)	1 (25.0)	0	0	4

^a^TTE: transthoracic echocardiography.

^b^KPSC: Kaiser Permanente Southern California.

^c^VHD: valvular heart disease.

**Table 6:** Severity of pulmonic stenosis captured in the 1,225,270 TTE^a^ reports in the KPSC^b^ health care system during 2011-2022 by VHD^c^, sex, race/ethnicity, and age at TTE time.

Characteristics		Severity of detected VHD				
		Mild/mild to moderate, n (%)	Moderate/moderate to severe, n (%)	Severe/very severe, n (%)	Unknown severity, n (%)	Total, N
**Sex**						
	Female	1063 (76.0)	190 (13.6)	28 (2.0)	118 (8.4)	1399
	Male	855 (72.1)	148 (12.5)	42 (3.5)	140 (11.8)	1185
**Age group (years)**						
	18-49	1182 (74.3)	245 (15.4)	53 (3.4)	111 (7.0)	1591
	50-64	356 (75.6)	58 (12.3)	8 (1.7)	49 (10.4)	471
	65-79	273 (71.9)	28 (7.3)	2 (0.5)	77 (20.3)	380
	≥80	1071 (74.8)	8 (5.6)	7 (4.9)	21 (14.7)	143
**Race/ethnicity**						
	Non-Hispanic White	742 (75.1)	104 (10.5)	27 (2.7)	115 (11.6)	988
	Non-Hispanic Black	134 (69.4)	29 (15.0)	3 (1.6)	27 (14.0)	192
	Hispanic	836 (74.2)	157 (14.0)	34 (3.0)	95 (8.5)	1122
	Non-Hispanic Asian/Pacific Islander	153 (73.6)	34 (16.3)	6 (3.0)	15 (7.2)	208
	Non-Hispanic Native American	7 (100.0)	0	0	0	7
	Multiple	8 (66.7)	2 (16.6)	0	2 (16.7)	12
	Other/unknown	38 (69.1)	13 (23.6)	0	4 (7.3)	55

^a^TTE: transthoracic echocardiography.

^b^KPSC: Kaiser Permanente Southern California.

^c^VHD: valvular heart disease.

**Table 7:** Severity of aortic regurgitation captured in the 1,225,270 TTE^a^ reports in the KPSC^b^ health care system during 2011-2022 by VHD^c^, sex, race/ethnicity, and age at TTE time.

Characteristics			Severity of detected VHD									
			Trace/trace to mild, n (%)		Mild/mild to moderate, n (%)		Moderate/moderate to severe, n (%)	Severe/very severe, n (%)		Unknown severity, n (%)		Total, N
**Sex**												
	Female	65,757 (40.1)		80,979 (50.6)		12,159 (7.6)		746 (0.5)	387 (0.2)		160,028	
	Male	76,036 (40.1)		92,242 (49.9)		14,507 (7.9)		1891 (1.0)	408 (0.2)		185,084	
**Age group (years)**												
	18-49	12,983 (53.8)		8250 (34.2)		2133 (7.9)		612 (2.5)	131 (0.5)		24,109	
	50-64	30,951 (49.9)		25,727 (41.4)		4499 (7.3)		791 (1.3)	179 (0.3)		62,147	
	65-79	64,739 (40.3)		79,578 (50.7)		11,329 (7.3)		853 (0.5)	331 (0.2)		156,830	
	≥80	33,121 (32.5)		59,666 (58.5)		8707 (8.6)		381 (0.4)	154 (0.2)		102,029	
**Race/ethnicity**												
	Non-Hispanic White	70,983 (40.6)		89,302 (51.1)		12,985 (7.5)		1083 (0.6)	339 (0.2)		174,692	
	Non-Hispanic Black	15,327 (41.5)		18,005 (48.6)		3291 (8.9)		349 (0.9)	75 (0.2)		37,047	
	Hispanic	37,552 (43.7)		37,628 (47.9)		6393 (7.4)		798 (0.9)	253 (0.3)		86,046	
	Non-Hispanic Asian/Pacific Islander	15,897 (37.3)		22,612 (53.0)		3669 (8.6)		362 (0.9)	117 (0.3)		42,657	
	Non-Hispanic Native American	271 (43.8)		293 (47.6)		53 (8.6)		3 (0.5)	0		620	
	Multiple	250 (46.7)		240 (44.9)		37 (7.0)		6 (1.1)	2 (0.4)		535	
	Other/unknown	1514 (43.1)		1719 (48.9)		240 (6.9)		36 (1.0)	9 (0.3)		3518	

^a^TTE: transthoracic echocardiography.

^b^KPSC: Kaiser Permanente Southern California.

^c^VHD: valvular heart disease.

**Table 8:** Severity of mitral regurgitation captured in the 1,225,270 TTE^a^ reports in the KPSC^b^ health care system during 2011-2022 by VHD^c^, sex, race/ethnicity, and age at TTE time.

Characteristics			Severity of detected VHD								
			Trace/trace to mild, n (%)	Mild/mild to moderate, n (%)		Moderate/moderate to severe, n (%)	Severe/very severe, n (%)		Unknown severity, n (%)		Total, N
**Sex**											
	Female	192,906 (48.3)		165,915 (41.6)	34,626 (8.7)		5519 (1.4)	613 (0.2)		399,579	
	Male	194,999 (48.5)		167,274 (41.5)	33,033 (8.2)		6421 (1.6)	780 (0.2)		402,507	
**Age group (years)**											
	18-49	79,986 (71.0)		27,457 (24.4)	3967 (3.5)		1082 (1.0)	162 (0.1)		112,654	
	50-64	109,278 (57.2)		67,784 (35.5)	11,095 (5.8)		2546 (1.3)	328 (0.2)		191,031	
	65-79	147,315 (44.8)		147,873 (44.9)	28,779 (8.7)		4982 (1.5)	608 (0.2)		329,557	
	≥80	51,336 (30.4)		90,081 (53.3)	23,819 (14.1)		3330(2.0)	295 (0.2)		168,861	
**Race/ethnicity**											
	Non-Hispanic White	185,392 (46.7)		170,099 (42.8)	35,180 (8.9)		6057 (1.5)	653 (0.2)		397,381	
	Non-Hispanic Black	40,623 (44.2)		40,065 (43.6)	9404 (10.3)		1746 (1.9)	166 (0.2)		92,004	
	Hispanic	113,637 (52.4)		81,306 (38.2)	14949 (7.0)		2648 (1.2)	353 (0.2)		212,893	
	Non-Hispanic Asian/Pacific Islander	40837 (47.0)		37239 (49.9)	7391 (8.5)		1313 (1.5)	198 (0.2)		86,978	
	Non-Hispanic Native American	847 (51.2)		651 (45.1)	127 (7.7)		24 (1.5)	4 (0.2)		1653	
	Multiple	901 (54.8)		585 (35.2)	132 (8.1)		30 (1.8)	4 (0.2)		1646	
	Other/unknown	5678 (59.4)		3256 (34.1)	477 (5.0)		122 (1.3)	15 (0.2)		9548	

^a^TTE: transthoracic echocardiography.

^b^KPSC: Kaiser Permanente Southern California.

^c^VHD: valvular heart disease.

**Table 9:** Severity of tricuspid regurgitation captured in the 1,225,270 TTE^a^ reports in the KPSC^b^ health care system during 2011-2022 by VHD^c^, sex, race/ethnicity, and age at TTE time.

Characteristics		Severity of detected VHD					
		Trace/trace to mild, n (%)	Mild/mild to moderate, n (%)	Moderate/moderate to severe, n (%)	Severe/very severe, n (%)	Unknown severity, n (%)	Total, N
**Sex**							
	Female	222,203 (48.3)	186,163 (40.5)	43,254 (9.4)	8131 (1.8)	339 (0.1)	460,090
	Male	239,533 (54.0)	170,161 (38.3)	29,393 (6.6)	4409 (1.0)	359 (0.1)	621,237
**Age group (years)**							
	18-49	97,093 (68.9)	38,822 (27.6)	4047 (2.9)	811 (0.6)	130 (0.1)	140,903
	50-64	129,591 (60.5)	73,292 (34.2)	9632 (4.5)	1698 (0.8)	175 (0.1)	214,388
	65-79	175,882 (48.1)	154,848 (42.4)	29,655 (8.1)	4849 (1.3)	286 (0.1)	365,520
	≥80	59,181 (32.3)	89,370 (48.8)	29,314 (16.0)	5182 (2.8)	107 (0.1)	183,154
**Race/ethnicity**							
	Non-Hispanic White	223,743 (50.9)	174,650 (39.7)	35,200 (8.0)	5438 (1.2)	314 (0.1)	439,345
	Non-Hispanic Black	46,610 (43.7)	45,674 (42.9)	11,694 (10.9)	2449 (2.3)	119 (0.1)	106,546
	Hispanic	134,122 (54.5)	91,627 (37.2)	16,949 (6.9)	3167 (1.3)	186 (0.1)	24,051
	Non-Hispanic Asian/Pacific Islander	48,438 (49.7)	39,461 (40.5)	8122 (8.3)	1362 (1.4)	71 (0.1)	97,454
	Non-Hispanic Native American	1018 (55.7)	669 (36.6)	115 (6.3)	24 (1.3)	1 (0.1)	1827
	Multiple	1083 (57.5)	658 (34.9)	113 (6.0)	29 (1.5)	1 (0.1)	1884
	Other/unknown	6733 (62.0)	3593 (33.1)	455 (4.2)	71 (0.7)	6 (0.1)	10,858

^a^TTE: transthoracic echocardiography.

^b^KPSC: Kaiser Permanente Southern California.

^c^VHD: valvular heart disease.

**Table 10:** Severity of pulmonic regurgitation captured in the 1,225,270 TTE^a^ reports in the KPSC^b^ health care system during 2011-2022 by VHD^c^, sex, race/ethnicity, and age at TTE time.

Characteristics		Severity of detected VHD					
		Trace/trace to mild, n (%)	Mild/mild to moderate, n (%)	Moderate/moderate to severe, n (%)	Severe/very severe, n (%)	Unknown severity, n (%)	Total, N
**Sex**							
	Female	99,962 (71.7)	36,513 (26.1)	2649 (1.9)	251 (0.2)	153 (0.1)	139,528
	Male	106,918 (72.5)	39,871 (27.1)	2791 (1.1)	211 (0.1)	129 (0.1)	147,369
**Age group (years)**							
	18-49	37,498 (79.5)	8574 (18.1)	734 (1.6)	294 (0.6)	71 (0.2)	47,171
	50-64	50,104 (77.7)	13,451 (20.9)	783 (1.2)	71 (0.1)	67 (0.1)	64,476
	65-79	80,120 (70.0)	32,104 (28.0)	2050 (1.8)	70 (0.1)	107 (0.1)	11,4451
	≥80	36,611 (60.2)	22,255 (36.6)	1875 (3.1)	27 (0.0)	37 (0.1)	60,850
**Race/ethnicity**							
	Non-Hispanic White	96,894 (71.9)	35,236 (26.2)	2314 (1.7)	184 (0.1)	110 (0.1)	134,738
	Non-Hispanic Black	22,603 (65.0)	11,185 (32.1)	946 (2.7)	51 (0.2)	38 (0.1)	34,823
	Hispanic	57,233 (74.0)	18,520 (24.8)	1308(1.7)	175 (0.2)	88 (0.1)	77,324
	Non-Hispanic Asian/Pacific Islander	23,854 (67.9)	10,391 (29.5)	822 (2.4)	46 (0.1)	40 (0.1)	35,153
	Non-Hispanic Native American	436 (73.1)	152 (25.5)	7 (1.2)	0	1 (0.2)	2025
	Multiple	490 (75.6)	147 (22.7)	11 (1.7)	0	0	648
	Other/unknown	2823 (78.0)	753 (20.9)	34 (0.9)	6 (0.2)	5 (0.1)	3621

^a^TTE: transthoracic echocardiography.

^b^KPSC: Kaiser Permanente Southern California.

^c^VHD: valvular heart disease.

## Discussion

### Principal Findings

In this study, we developed a computerized algorithm to identify the presence/absence and the severity of stenosis and regurgitation of the 4 heart valves (aortic, mitral, tricuspid, and pulmonic) from reports of routinely performed TTEs. This algorithm yielded high accuracy in extracting information, except for a few severity groups due to their small numbers. This process was successfully implemented in a large integrated health care system to estimate the frequencies of VHD described in the TTE reports among a demographically diverse population.

### Comparison With Prior Work

Echocardiography is the primary imaging technique for evaluating the severity of VHD. Incorporating an NLP algorithm developed to extract information about valvular lesion severity from unstructured echocardiogram reports allows the identification of patients with VHD across a large population. This is useful because the frequency of surveillance imaging is dependent on the severity of the valvular lesion. Specifically, patients with mild valvular lesions typically require imaging every 3-5 years, those with moderate lesions need evaluations every 1-2 years, and those with severe lesions need evaluations every 6-12 months [28]. Identifying and categorizing patients with VHD at a population level ensures that all patients receive timely and adequate follow-up.

The performance of the algorithms reported in this study was comparable with those reported in previous studies [6,18,19]. In line with findings from previous studies [6,18,19], we observed that the percentage of VHD increases with patient age [9]. VHD affected both sexes, although certain conditions showed sex-specific patterns. Aortic regurgitation was more commonly observed in males, a finding that aligns with other studies, indicating a male predominance of aortic regurgitation [4]. Conversely, tricuspid regurgitation was more commonly observed in females in this population. Further research into the incidence, prevalence, and associated risk factors of these valvular lesions will enhance our understanding of the causes behind the observed sex differences [29].

Recent studies have attempted to extract stenosis and regurgitation from echocardiography reports [6,17-19]. Solomon et al [6] focused primarily on the extraction of aortic stenosis and a few continuous measurements. Although Nath et al [18] and Dong et al [19] attempted to retrieve stenosis and regurgitation of heart valves, their performances were not assessed for each condition independently, and neither of the authors evaluated performance by severity of illness. Even for the combined evaluation, Dong et al [19] reported low performance for both the precision and recall of identifying stenosis of the 4 heart valves. The approach taken by this study has several advantages. First, part of our training and validation samples included TTE reports of potential patients diagnosed with mitral, tricuspid, and pulmonic stenosis. Therefore, the samples included a fair number of patients with these relatively rare conditions, which allowed the computerized algorithm to train/recognize corresponding potential patterns. Second, our study evaluated each case of stenosis or regurgitation independently. However, the performance of some severity levels needs cautious interpretation due to few cases and small validation samples. A larger sample and additional validations based on external data sets in future work can yield more robust performance and strengthen the evidence for the algorithm’s utility and robustness in real-world clinical settings.

### Strengths and Limitations

A key feature of our algorithm is its ability to extract important elements from written echocardiogram reports and convert them into structured data elements. This capability enables a health care system to provide true population care by tracking the number of patients with varying degrees of valvular lesions. By doing so, the health care system can ensure that surveillance monitoring and follow-up appointments are appropriately scheduled. This integration was a crucial consideration for this study. It will be a focus of future work, as it enhances the practical utility and adoption of the algorithm in clinical settings. In addition to extracting severity, future work will also enhance the computerized algorithm to retrieve other VHD-related measurements [19] to facilitate patient care and management.

Research of heart valve conditions based on diagnosis codes only may be impacted by the inaccuracy of coding, especially for minority populations. Crousillat et al [7] showed that diagnosis codes for aortic stenosis are less accurate for racial and ethnic minorities and less severe stages of the disease and, therefore, cannot be used to evaluate observed care disparities. This issue is likely to be alleviated by the application of NLP to TTE reports. Solomon et al [6] demonstrated that NLP application captures 35.4% more aortic stenosis compared to diagnosis code identification. Future studies are needed to understand and mitigate recently identified VHD care disparities and improve outcomes for patients [28].

This study primarily used a rule-based approach for NLP. Transformer-based models, such as bidirectional encoder representations from transformers (BERT) [30], have gained popularity in recent years in clinical research involving NLP. These large language models can effectively capture the text’s intricate relationships via word embedding representation and attention mechanism and, therefore, are capable of analyzing information from unstructured notes in the health care domain more accurately [31-34]. Future research may integrate these sophisticated machine learning or deep learning language models into NLP algorithms to further boost performance and to handle the complexity of medical language more effectively.

Our study acknowledges several limitations. First, the completeness and accuracy of the extracted information were dependent on the information documented in the TTE reports. Incomplete or inaccurate documentation could lead to misclassification. Despite our efforts to correct misspelled words, there could be additional unidentified errors. Second, the templated formats of echocardiography reports could limit the diversity and specificity of language used, potentially affecting the algorithm’s sensitivity. Third, although our training process was quite comprehensive and included a relatively large number of notes, the rules and lexicons developed from our training data sets were still not highly comprehensive. For example, the severity of mitral stenosis in “moderate to borderline severe calcific mitral stenosis” should be “moderate to severe.” However, the algorithm identified it as “severe” because of the additional word “borderline” prior to “severe.” Therefore, more samples could be used to enhance the rules and lexicons in the future, especially for rare conditions (mitral, tricuspid, and pulmonic stenosis). Fourth, numerous abbreviations in the study used terms with multiple meanings, which complicated the identification process. For instance, the word “as” could mean “aortic stenosis”; “ms” could mean “mitral stenosis” or “millisecond,” a time unit used for velocity measurements; and “tr” could mean “trace” or “tricuspid regurgitation.” Although we applied a set of rules to determine the exclusion of used abbreviated terms, the algorithm could still potentially misuse the meaning of the abbreviation of these terms. Fifth, if a severity term was found to appear prior to a set of stenosis or regurgitation terms listed together (eg, mild as/ai/mr), our algorithm only assigned the severity to the first term, leading to incomplete labeling of the severity of other terms. Sixth, the TTE reports of patients with congenital valvular conditions frequently used the terms “systemic AV” and “subpulmonic AV,” which represented the morphologic tricuspid valve and morphologic mitral valve, respectively. However, the meanings of these 2 terms are different in patients with noncongenital disease. Our algorithm did not search for these 2 terms for patients with congenital valvular conditions, which could lead to potential misclassification of congenital valvular conditions. Although the population of patients with congenital VHDs is very small, future work can modify the algorithm to improve the performance for congenital valvular conditions. Lastly, although the NLP algorithm in this study was trained by a large number of annotated echocardiogram reports from the KPSC, algorithm may need to be adjusted before it is implemented in other health care organizations, due to variations in report formatting and reporting requirements. Typically, reformatting echocardiogram reports before implementing the algorithm can enhance its adaptability. When feasible, users may also consider retraining the algorithm using notes specific to the organization for improved performance. However, the complexity of our NLP algorithm and process might limit its adoption in settings without specialized expertise in NLP or access to similar resources for algorithm development and validation [35].

### Conclusion

The computerized algorithm developed can effectively identify heart valve stenosis and regurgitation and the severity of valvular involvement. This algorithm has potential applications in clinical research and patient cardiovascular care management. The computerized algorithm needs further adjustments to accommodate variations in the format and presentation of TTE reports when it is implemented in other health care organizations.

## Appendix

Multimedia Appendix 1 Examples of deidentified echocardiogram reports, search terms used for capturing heart valve stenosis and regurgitation, terms used for stenosis and regurgitation severity, corrections of mistyped words/terms used to search for heart valve stenosis and regurgitation and severity, sections of TTE reports used to define heart valve stenosis and regurgitation severity, terms used to search and exclude for history description of stenosis and regurgitation, detection frequency and severity of stenosis and regurgitation by valvular disease, severity of stenosis and regurgitation captured in TTE reports by valvular disease, sex, race/ethnicity, and age. TTE: transthoracic echocardiography.

Multimedia Appendix 2 High resolution version of Figure 3.

## Abbreviations

EHR: electronic health record
FN: false negative
FP: false positive
KPSC: Kaiser Permanente Southern California
NLP: natural language processing
PPV: positive predictive value
TP: true positive
TTE: transthoracic echocardiography
VHD: valvular heart disease
