# Supplementary material for: Identifying the Severity of Heart Valve Stenosis and Regurgitation Among a Diverse Population Within an Integrated Health Care System: Natural Language Processing Approach
Source: JMIR Cardio. 2024 Sep 30;8:e60503. doi: 10.2196/60503 (PMC11474122; doi:10.2196/60503)
Supplement: Multimedia Appendix 1 [file cardio_v8i1e60503_app1.docx]

Table S1. Examples of deidentified echocardiogram reports from the study period.

| Example 1 | **Transthoracic Echocardiography Report (TTE)**  **Demographics**  Patient Name         Gender  Medical Record Number          Date of Study  Accession Number           Referring Physician  Date of Birth          Sonographer  Age         Interpreting Physician  Procedure  Type of Study  TTE procedure: ECHOCARDIOGRAM TRANSTHORACIC (TTE)  Procedure Date  Date:       Start:  Technical Quality: Adequate  Indications: Cardiovascular Screening.  Patient Status: Outpatient  Conclusions  **Summary**  There is normal left ventricular size and systolic function (visually estimated EF  %). There is no hypertrophy or wall motion abnormalities. Normal right ventricular size and systolic function. There is no significant valvular disease.  No prior studies for comparison.  **M-Mode/2D Measurements & Calculations**  LV Diastolic Dimension:   cm LA volume/Index:   ml /   m^2  LV Systolic Dimension:   cm LVOT:   cm  LV Septum Diastolic:   cm    AO Root:   cm  LV PW Diastolic:   cm  EF% (Simpson's):   %           Ascending Aorta:   cm  Spectral and Color Doppler Measurements  AV Peak Vel:   m/s          MV Peak E-Wave:   m/s  AV Peak PG:   mmHg          MV Peak A-Wave:   m/s  AV Mean PG:   mmHg             MV E/A Ratio:  AV Area (Continuity):   cm^2 MV Peak Gradient:   mmHg  LVOT Peak Vel:   m/s  **Findings**  Left Ventricle  There is normal left ventricular size and systolic function (visually estimated EF   %). There is no hypertrophy or wall motion abnormalities. The diastolic function is normal.  The left ventricular ejection fraction based on Simpson's calculation is   %.  Right Ventricle  Normal right ventricular size and systolic function. TAPSE is normal at   cm.  There is inadequate tricuspid valve regurgitation to estimate the pulmonary artery pressure.  Left Atrium  Left atrium is normal in size. Left atrial volume index is   ml/m2.  Right Atrium  Right atrium is normal in size. The inferior vena cava is normal in diameter and collapses appropriately with inspiration suggestive of normal right atrial pressure.  Aorta  Aortic root and ascending aorta are normal in diameter.  Mitral Valve  Mitral valve is structurally normal. There is no evidence of significant valvular stenosis or insufficiency.  Aortic Valve  Aortic valve is trileaflet and opens well. There is no evidence of significant valvular stenosis or insufficiency.  Tricuspid Valve  The tricuspid valve is structurally normal. There is no significant tricuspid regurgitation or stenosis.  Pulmonic Valve  The pulmonic valve is structurally normal. There is no evidence of pulmonic valve stenosis.  No evidence of pulmonic regurgitation.  Pericardium and Pleura  No evidence of pericardial effusion. No evidence of pleural effusion.  **Signature**  Electronically signed by       MD on |
| --- | --- |
| Example 2 | **ECHOCARDIOGRAM REPORT**  PATIENT NAME:  MEDICAL RECORD NUMBER:  DATE OF BIRTH:  ECHO TECH:  TAPE NUMBER:  DATE OF PROCEDURE:  REFERRING PHYSICIAN:  INDICATION: EVAL PRESYNCOPE  **DIMENSIONS**  LV end systolic diameter:  cm (normal range  cm)  LV end diastolic diameter:  cm (normal range  cm)  LV IVS (D):   cm (normal range  cm)  LV PW (D):   cm (normal range  cm)  Left atrium (ES):  cm (normal range  cm)  Aortic root (ED):  cm (normal range  cm)  LVEF:  % (normal >  %)  **EXAM QUALITY:** Fair. Patient is in sinus rhythm during the study.  **FINDINGS**  LEFT VENTRICLE: LV size is normal. LV wall thickness is normal. Overall LV systolic function is normal with visually estimated ejection fraction of  %. There are no significant wall motion abnormalities. Diastolic function is normal.  LEFT ATRIUM: Left atrial size is normal.  RIGHT HEART: Right atrial size is normal. Right ventricular size is normal with normal systolic function. Inferior vena cava size is not dilated with normal respiratory collapse.  AORTIC VALVE: The aortic valve structure is trileaflet. There is mild eccentric aortic regurgitation. Aortic valve peak velocity is normal,  m/sec and peak gradient is not  significant. Left ventricular outflow tract peak velocity is normal.  MITRAL VALVE: The mitral valve structure is normal. There is no significant mitral regurgitation.  PULMONIC VALVE: The pulmonic valve structure is normal. There is no significant pulmonic regurgitation.  TRICUSPID VALVE: The tricuspid valve structure is normal. There is no significant tricuspid regurgitation. Can not estimate systolic pulmonary artery pressure.  PERICARDIUM: There is no significant pericardial effusion.  AORTA: Aortic root size is normal.  **CONCLUSIONS:**  1. Normal LV size & function.  2. Mild eccentric aortic regurgitation.  **Electronically signed by**:       MD on |

Table S2. Search terms used for capturing stenosis and regurgitation for each heart valve.

| Heart valve | Stenosis* | Regurgitation* |
| --- | --- | --- |
| aortic valve, av | aortic stenosis, aortic valve stenosis, as, avs | aortic regurgitation, aortic valve regurgitation, ar, avr, aortic insufficiency, aortic valve insufficiency, ai, avi |
| mitral valve, mv | mitral stenosis, mitral valve stenosis, ms, mvs | mitral regurgitation, mitral valve regurgitation, mr, mvr, mitral insufficiency, mitral valve insufficiency, mi, mvi |
| tricuspid valve, tv | tricuspid stenosis, tricuspid valve stenosis, ts, tvs | tricuspid regurgitation, tricuspid valve regurgitation, tr, tvr, tricuspid insufficiency, tricuspid valve insufficiency, ti, tvi |
| pulmonic valve, pulmonary valve, pv | pulmonic stenosis, pulmonary stenosis, pulmonic valve stenosis, pulmonary valve stenosis, ps, pvs | pulmonic regurgitation, pulmonary regurgitation, pulmonic regurgitation valve regurgitation, pulmonary regurgitation valve regurgitation, pr, pvr, pulmonic insufficiency, pulmonary insufficiency, pulmonic valve insufficiency, , pulmonary valve insufficiency, pi, pvi |

* The number of words between heart valve terms and the stenosis and regurgitation terms is limited to 6 to positive stenosis and regurgitation, respectively. Also search lemma variants (plural form), where applicable.

Table S3. Terms used stenosis and regurgitation severity.

| Severity category | Keywords/terms |
| --- | --- |
| Prosthetic | prosthetic, prosthesis, bioprosthetic, bioprosthesis, mechanic, biologic, well-seated, porcine, bovine, valve replacement, valve repair, valve ring implant, valve annuloplasty ring, edwards sapien, corevalve, st jude, hancock, trifecta, freestyle, melody |
| Sclerosis^£^ | sclerosis |
| Trace^¶^ | trace, trivial, physiologic |
| Trace to mild^¶^ | trace to mild, trace/mild, trace-mild, trace~mild |
| Mild | mild, 1+^¶^, one plus |
| Mild to moderate | mild to moderate, mild/moderate, mild-moderate, mild~moderate, 2+^¶^, tow plus |
| Moderate | moderate, mod |
| Moderate to severe | moderate to severe, moderate/severe, moderate-severe, moderate~severe, 3+^¶^, three plus |
| Severe | severe, 4+^¶^, four plus |
| Very severe | very severe, critical severe, significant severe, extremely severe, severe significantly, very significant, very critical, wide open^¶^ |

* Also search lemma variants (adjective, adverb, plural form) and abbreviations of these words, where applicable.

£ only apply for aortic stenosis.

¶ only apply for regurgitation

Table S4. Corrections of mistyped or concatenating words/terms used for searching heart valve stenosis and regurgitation and severity in TTE reports.

| Mistyping or concatenating words/terms | Corrected words/terms |
| --- | --- |
| ortic, aor tic, sortic, aortiuc, vaortic | aortic |
| aortivvalve | aortic valve |
| noortic, noa ortic, | no aortic |
| sigaortic | significant aortic |
| mi tral, miral, m itral, mitrall, mittral, imtral | mitral |
| tr icuspid, tri cuspid, ricuspid, tricu spid, | tricuspid |
| pulmoni c, pulmon nic, pulm, lmonic, ulmonic, puln, puolm, dpulm | pulmonic |
| pumonary, pulmnary, pulmonmary, pulmoary, ulmonary, pulmonar, puimonary, pulmomary, pulmoany, pumlmonary, pulomonary, pulmona, piulmonary, pulonary, pulmonry, pulmoanry, pulnmonary, pulmo0nary, pulmonnary, pulmomnary, pulmonaty, pulmonart, lmonary, pulmarary, pulomonay, pulmonay, plmonary, pulmnoary | pulmonary |
| vale, vlave, | valve |
| stensois, stenosi, stenos, stensosis, | stenosis |
| gurgitation, regurg, regurgitaton, regurgtiation, regurgit, regurgitaiton, regurgiation, regurigation, regugitation, regurgitgation, regurgitration, reguration, regurgetation | regurgitation |
| mitral r | mitral regurgitation |
| mr ot ms | mr or ms |
| prothetic, prosthestic, proathetic, | prosthetic |
| prothesis, prostehesis | prosthesis |
| prosthesiswith | Prosthesis with |
| bioprothetic, bioprosthtic, bio prosthtic, biprosthetic, bio-prosthetic, biosprosthetic, bioprostetic, bioprostethic, bioprosethetic, bioprsthetic, bioprosthetric, bioprosthtic, bioprostheticl, bioprosthetic, bioprosthet9c, bioprostatic, bioprosthethic, bioposthetic, biop | bioprosthetic |
| bioprothesis, bio prothesis, boprosthesis, bioprostehsis, bioprsthesis, bio prsthesis, bio-prosthesis, bioprpsthesis, biorprosthesis | bioprosthesis |
| sleriosis, sclerois, scleraosis | sclerosis |
| sev, sevefre, severte, evere, devere, sevre, sever,sveere, severee | severe |
| severeaortic | Severe aortic |
| nonsevere | non severe |
| criticalm, ciritical | critical |
| modsevere | moderate severe |
| mo derate, oderate, morate, oderate, mo, mioderate, modaret, modrate, mioderate, mode, modeate, moderarte, moderte, moerate, mooderate, modera te, modest, moderator, mmoderate, modearate , moderaet, moderaste , moderte, modest, modetate, modrate, mokderate, modferate, modd, mderate, modeate, mioderate, modrerate, mdoerate, moderatea, moderato | moderate |
| andmoderate | and moderate |
| moderteaortic | moderate aortic |
| moderateto | moderate to |
| functionmod | function moderate |
| mold to omod, mil/dmod | mild to moderate |
| regurgitationmild | regurgitation mild |
| miid, mikld, mid, midl, ild, mid, mil, mld, miild, mile, mlild, mi ld, mila, nild, nmild, mld, midl, milk, mils, miuld, mild, miuld, mlid, mil, miled, milf, milid, milld, miold, miid, mlid, milt, iild, mil d, mil;d, m\ild, milc, mmild, mold, mils  , mil;d, <ild, mi;d, milf, mlld, nild, tmild, fmild, mill, mold, mile | mild |
| withmild | with mild |
| seenmild | seen mild |
| functionmild | Function mild |
| visulaziationmild | visualization mild |
| valvemild | valve mild |
| appeamild | appear mild |
| ismildo, ismoid | is mild |
| nomild | no mild |
| tracemild | trace mild |
| tracce, tracd, tracea, traces, traced, tracet, trade, trasce, travce, traxce, traxe, trce, twace , trac e, tracee, treace, ntrace, rtrace, tarce, tr ace, tra, tace, traec, rrace, tracer, ytace, ttrace, ttace, trqce, trrace , trtace, tracfe, tracxe, trae, trance, triace, tracel, traace, grace, trac, trave, trcae, mtrace, tracing, strtrace, tracr, traqce, tra ce, tract, tracec, tracev, tracae, tracew, tracs, trice, tracd, tracve, trxw, tracde, traece, trash, trqace | trace |
| istrace, , istace | is trace |
| functiontr, functiontrace, functrace | function trace |
| Tracemitral | trace mitral |
| Tracepulmonic | trace pulmonic |
| Withtrace | with trace |
| Valvetrace | valve trace |
| trivia, trivail, trivak, trvial, tivial, trivial, trival, tivial, tr ivial, trivail, tivial, triv,  triival, trivvial, traivial, tivail, tri, trival, tivial, trivi, triviai, trvivial, ivial, | trivial |
| Istrivial | is trivial |
| phyrio, physio | physiologic |
| witjout, withotu, withouit, witout, wit hout, witho ut, w ithout, wi thout, withou t, withour, with out, wihtout, withouy, withoutwitout, without, withoutwitjout, withoutwitou, wtihout, w/o, wihout, ithout | without |
| Valvetithout | valve without |
| isno, i sno, ino, | is no |
| Noignificant | no significant |
| sig, sign, sig nificant, signif, s ignificant, signficant, signfiicant, signifcant, signifciant, signifiant, signifincant, | significant |
| Tracemildno | trace mild no |
| evide | evidence |
| evid of | evidence of |
| Mechavr | mechanical avr |

Table S5. The sections and subsections of TTE Echo reports used to define heart valve stenosis and regurgitation severity.

| TTE report section | Aortic valve | Mitral valve | Tricuspid valve | Pulmonic valve |
| --- | --- | --- | --- | --- |
| Conclusion/Summary | x | x | x | x |
| Findings/results: Aortic valve | x |  |  |  |
| Findings/results: Mitral valve |  | x |  |  |
| Findings/results: Tricuspid valve |  |  | x |  |
| Findings/results: Pulmonic valve |  |  |  | x |
| Impression | x | x | x | x |

Table S6. Terms used to search and exclude for history description of stenosis and regurgitation.

| Term | last study, previous study, prior study, last tte, previous tte, prior tte, last echo, previous echo, prior echo, history of |
| --- | --- |
| Date | study from [date], study done on [date], tte from [date], tte done on [date], echo from [date], echo done on [date] |

[date]: 1) included various date format, such as 1/2011, 6/5/2007, etc. 2) The date was prior the current TTE performed date.

Table S7. Detection frequency and severity of stenosis and regurgitation by valve disease based on 1,225,270 TTE reports performed in the KPSC setting during 2011-2022. Reported as N (column %).

| Conditions detected in TTE reports (n,%) | Stenosis | | | | Regurgitation | | | | | |
| --- | --- | --- | --- | --- | --- | --- | --- | --- | --- | --- |
|  | Aortic valve | Mitral valve | Tricuspid valve | Pulmonic valve | | Aortic valve | Mitral valve | Tricuspid valve | | Pulmonic valve |
| Prosthetic | 50507 (4.12) | 22656 (1.85) | 1685 (0.14) | 1767 (0.14) | | 50507 (4.12) | 22656 (1.85) | | 1685 (0.14) | 1767 (0.14) |
| Sclerosis | 286784 (23.40) | - | - | - | | - | - | | - | - |
| No/no evidence | 776679 (63.40) | 1182368 (96.50) | 1223188 (98.83) | 1220918 (99.65) | | 829648 (67.71) | 400511 (32.69) | | 319620 (26.09) | 936600 (76.44) |
| Valve disease detected^¶^ | 111300 (9.08) | 20246 (1.65) | 397 (0.03) | 2585 (0.21) | | 345115 (28.17) | 802103 (65.46) | | 903965 (73.78) | 286903 (23.42) |
| Trace | - | - | - | - | 133423 (38.7) | | 367737 (45.9) | 439725 (48.6) | | 199346 (69.5) |
| Trace to mild | - | - | - | - | 8371 (2.4) | | 20178 (2.5) | 22022 (2.4) | | 4887 (1.7) |
| Mild | 49845 (44.8) | 11078 (54.7) | 196 (49.4) | 1758 (68.0) | 146202 (42.4) | | 280238 (34.9) | 309498 (34.2) | | 71097 (24.8) |
| Mild to moderate | 9125 (8.2) | 2183 (10.8) | 67 (16.9) | 160 (6.2) | 27019 (7.8) | | 52957 (6.6) | 46834 (5.2) | | 5287 (1.8) |
| Moderate | 24517 (22.0) | 3995 (19.7) | 45 (11.3) | 301 (11.6) | 22912 (6.6) | | 51847 (6.5) | 57429 (6.4) | | 4998 (1.7) |
| Moderate to severe | 7615 (6.8) | 901 (4.5) | 11 (2.8) | 38 (1.5) | 3756 (1.1) | | 15813 (2.0) | 15219 (1.7) | | 444 (0.2) |
| Severe | 17091 (15.4) | 1216 (6.0) | 24 (6.1) | 66 (2.6) | 2624 (0.8) | | 11906 (1.5) | 12512 (1.4) | | 0 (0.0) |
| Very severe | 1199 (1.1) | 11 (0.1) | 0 (0.0) | 4 (0.2) | 13 (0.0) | | 34 (0.0) | 0 (0.0) | | 0 (0.0) |
| Unknown severity | 1908 (1.7) | 862 (4.3) | 54 (13.6) | 258 (10.0) | 795 (0.2) | | 1393 (0.2) | 698 (0.1) | | 282 (0.1) |

TTE: Transthoracic echocardiogram

KPSC: Kaiser Permanente Southern California

¶ percentage among the severity group

Table S8. The severity of stenosis and regurgitation captured in the 1,225,270 TTE reports performed at KPSC during 2011-2022 by valve disease, sex, race/ethnicity, and age at the TTE performed time. Reported as N (row %).

|  | **Severity of detected heart valve disease** | | | | | | | | | |
| --- | --- | --- | --- | --- | --- | --- | --- | --- | --- | --- |
|  | Trace | Trace to mild | Mild | Mild to moderate | Moderate | Moderate to severe | Severe | Very severe | Unknown severity | All |
| **Aortic stenosis** |  |  |  |  |  |  |  |  |  |  |
| Sex |  |  |  |  |  |  |  |  |  |  |
| Female | - | - | 23784 (46.8) | 4088 (8.0) | 10739 (21.1) | 3228 (6.4) | 7461 (14.7) | 671 (1.3) | 887 (1.7) | 50858 |
| Male | - | - | 26061 (43.1) | 5037 (8.3) | 13777 (22.8) | 4386 (7.3) | 9640 (15.9) | 528 (0.9) | 1021 (1.7) | 60440 |
| Age group |  |  |  |  |  |  |  |  |  |  |
| 18-49 | - | - | 1581 (51.3) | 216 (7.0) | 718 (23.3) | 120 (3.9) | 287 (9.3) | 15 (0.5) | 147 (4.8) | 3084 |
| 50-64 | - | - | 6206 (48.1) | 934 (7.2) | 2659 (20.6) | 708 (5.5) | 1955 (15.2) | 143 (1.1) | 290 (2.3) | 12895 |
| 65-79 | - | - | 23690 (46.9) | 4165 (8.2) | 11004 (21.8) | 3251 (6.4) | 7678 (17.2) | 464 (0.9) | 803 (1.6) | 50548 |
| 80+ | - | - | 18368 (41.0) | 3810 (8.5) | 10136 (22.6) | 3536 (7.9) | 7678 (17.2) | 577 (1.3) | 668 (1.5) | 44773 |
| Race/ethnicity |  |  |  |  |  |  |  |  |  |  |
| Non-Hispanic white | - | - | 28652 (43.3) | 5387 (8.2) | 14816 (22.4) | 4796 (7.3) | 10731 (16.2) | 712 (1.1) | 1019 (1.5) | 66113 |
| Non-Hispanic black | - | - | 4174 (47.7) | 743 (8.5) | 1922 (22.0) | 551 (6.3) | 1116 (12.8) | 81 (0.9) | 167 (1.9) | 8754 |
| Hispanic | - | - | 11868 (45.0) | 2140 (8.1) | 5728 (21.7) | 1724 (6.5) | 4043 (15.3) | 333 (1.3) | 533 (2.0) | 26368 |
| Non-Hispanic  Asian/pacific islander | - | - | 4560 (52.0) | 763 (8.7) | 1738 (19.8) | 490 (5.6) | 1006 (11.5) | 59 (0.7) | 167 (1.9) | 8783 |
| Non-Hispanic Native  American | - | - | 97 (44.3) | 19 (8.7) | 44 (20.1) | 8 (3.7) | 42 (19.2) | 4 (1.8) | 5 (2.3) | 219 |
| Multiple | - | - | 61 (43.6) | 10 (7.1) | 38 (27.1) | 3 (2.1) | 20 (14.3) | 1 (0.7) | 7 (5.0) | 140 |
| Other/Unknown | - | - | 433 (46.9) | 63 (6.8) | 231 (25.0) | 43 (4.7) | 133 (14.4) | 10 (1.1) | 10 (1.1) | 923 |
| **Aortic regurgitation** |  |  |  |  |  |  |  |  |  |  |
| Sex |  |  |  |  |  |  |  |  |  |  |
| Female | 61858 (38.7) | 3899 (2.4) | 67754 (42.3) | 13225 (8.3) | 10776 (6.7) | 1383 (0.9) | 739 (0.5) | 7 (0.0) | 387 (0.2) | 160028 |
| Male | 71564 (38.7) | 4472 (2.4) | 78448 (42.4) | 13794 (7.5) | 12134 (6.6) | 2373 (1.3) | 1885 (1.0) | 6 (0.0) | 408 (0.2) | 185084 |
| Age group |  |  |  |  |  |  |  |  |  |  |
| 18-49 | 12257 (50.8) | 726 (3.0) | 6734 (27.9) | 1516 (6.3) | 1608 (6.7) | 525 (2.2) | 611 (2.5) | 1 (0.0) | 131 (0.5) | 24109 |
| 50-64 | 29362 (47.3) | 1589 (2.6) | 22000 (35.4) | 3727 (6.0) | 3648 (5.9) | 851 (1.4) | 789 (1.3) | 2 (0.0) | 179 (0.3) | 62147 |
| 65-79 | 60783 (38.8) | 3956 (2.5) | 67482 (43.0) | 12096 (7.7) | 9834 (6.3) | 1495 (1.0) | 846 (0.5) | 7 (0.0) | 331 (0.2) | 156830 |
| 80+ | 31021 (30.4) | 2100 (2.1) | 49986 (49.0) | 9680 (9.5) | 7822 (7.7) | 885 (0.9) | 378 (0.4) | 3 (0.0) | 154 (0.2) | 102029 |
| Race/ethnicity |  |  |  |  |  |  |  |  |  |  |
| Non-Hispanic white | 66754 (38.2) | 4229 (2.4) | 75308 (43.1) | 13994 (8.0) | 11261 (6.5) | 1724 (1.0) | 1079 (0.6) | 4 (0.0) | 339 (0.2) | 174692 |
| Non-Hispanic black | 14550 (39.3) | 777 (2.1) | 15254 (41.2) | 2751 (7.4) | 2806 (7.6) | 485 (1.3) | 348 (0.9) | 1 (0.0) | 75 (0.2) | 37047 |
| Hispanic | 35341 (41.1) | 2211 (2.6) | 34877 (40.5) | 2751 (7.4) | 5422 (6.3) | 971 (1.1) | 791 (0.9) | 7 (0.0) | 253 (0.3) | 86046 |
| Non-Hispanic  Asian/pacific islander | 14852 (34.8) | 1045 (2.5) | 18844 (44.2) | 3768 (8.8) | 3147 (7.4) | 522 (1.2) | 361 (0.9) | 1 (0.0) | 117 (0.3) | 42657 |
| Non-Hispanic Native  American | 257 (41.5) | 14 (2.3) | 240 (38.7) | 53 (8.9) | 47 (7.6) | 6 (1.0) | 3 (0.5) | 0 (0.0) | 0 (0.0) | 620 |
| Multiple | 242 (45.2) | 8 (1.5) | 202 (37.8) | 38 (7.1) | 26 (4.9) | 11 (2.1) | 6 (1.1) | 0 (0.0) | 2 (0.4) | 535 |
| Other/Unknown | 1427 (40.6) | 87 (2.5) | 1477 (42.0) | 242 (6.9) | 203 (5.8) | 37 (1.1) | 36 (1.0) | 0 (0.0) | 9 (0.3) | 3518 |
| **Mitral stenosis** |  |  |  |  |  |  |  |  |  |  |
| Sex |  |  |  |  |  |  |  |  |  |  |
| Female | - | - | 7536 (53.3) | 1562 (11.1) | 2850 (20.2) | 675 (4.8) | 923 (6.5) | 6 (0.0) | 579 (4.1) | 14130 |
| Male | - | - | 3542 (57.9) | 621 (10.2) | 1145 (18.7) | 226 (3.7) | 293 (4.8) | 5 (0.1) | 284 (4.6) | 6116 |
| Age group |  |  |  |  |  |  |  |  |  |  |
| 18-49 | - | - | 399 (41.1) | 115 (11.8) | 205 (21.1) | 70 (7.2) | 106 (10.9) | 1 (0.1) | 75 (7.7) | 971 |
| 50-64 | - | - | 1235 (45.3) | 271 (9.9) | 643 (23.6) | 164 (6.0) | 269 (9.9) | 1 (0.0) | 145 (5.3) | 2728 |
| 65-79 | - | - | 4727 (54.8) | 945 (11.0) | 1687 (19.5) | 390 (4.5) | 521 (6.0) | 6 (0.1) | 356 (4.1) | 8632 |
| 80+ | - | - | 4717 (59.6) | 852 (10.8) | 1460 (18.5) | 277 (3.5) | 320 (4.0) | 3 (0.0) | 286 (3.6) | 7915 |
| Race/ethnicity |  |  |  |  |  |  |  |  |  |  |
| Non-Hispanic white | - | - | 5969 (58.2) | 1090 (10.6) | 1965 (19.2) | 395 (3.9) | 464 (4.5) | 3 (0.0) | 375 (3.7) | 10261 |
| Non-Hispanic black | - | - | 937 (54.3) | 202 (11.7) | 304 (17.6) | 72 (4.4) | 123 (7.1) | 1 (0.1) | 84 (4.9) | 1725 |
| Hispanic | - | - | 3014 (52.0) | 638 (11.0) | 1168 (20.1) | 272 (4.7) | 413 (7.1) | 3 (0.1) | 293 (5.1) | 5801 |
| Non-Hispanic  Asian/pacific islander | - | - | 1047 (46.2) | 239 (10.5) | 522 (23.0) | 154 (6.8) | 202 (8.9) | 2 (0.1) | 102 (4.5) | 2268 |
| Non-Hispanic Native  American | - | - | 18 (51.4) | 2 (5.7) | 9 (25.7) | 0 (0.0) | 4 (11.4) | 0 (0.0) | 2 (5.7) | 35 |
| Multiple | - | - | 20 (71.4) | 1 (3.6) | 4 (14.3) | 2 (7.1) | 0 (0.0) | 0 (0.0) | 1 (3.6) | 28 |
| Other/Unknown | - | - | 73 (57.0) | 12 (9.4) | 23 (18.0) | 3 (2.3) | 10 (7.8) | 2 (1.6) | 5 (3.9) | 128 |
| **Mitral regurgitation** |  |  |  |  |  |  |  |  |  |  |
| Sex |  |  |  |  |  |  |  |  |  |  |
| Female | 182678 (45.7) | 10228 (2.6) | 138467 (34.7) | 27448 (6.9) | 26641 (6.7) | 7985 (2.0) | 5504 (1.4) | 15 (0.0) | 613 (0.2) | 399579 |
| Male | 185049 (46.0) | 9950 (2.5) | 141765 (35.2) | 25509 (6.3) | 25205 (6.3) | 7828 (1.9) | 6402 (1.6) | 19 (0.0) | 780 (0.2) | 402507 |
| Age group |  |  |  |  |  |  |  |  |  |  |
| 18-49 | 76721 (68.1) | 3265 (2.9) | 24458 (21.7) | 2999 (2.7) | 2917 (2.6) | 1050 (0.9) | 1075 (1.0) | 7 (0.0) | 162 (0.1) | 112654 |
| 50-64 | 103920 (54.4) | 5358 (2.8) | 59059 (30.9) | 8725 (4.6) | 8367 (4.4) | 2728 (1.4) | 2541 (1.3) | 5 (0.0) | 328 (0.2) | 191031 |
| 65-79 | 138927 (42.2) | 8388 (2.6) | 124236 (37.7) | 23637 (7.2) | 22160 (6.7) | 6619 (2.0) | 4971 (1.5) | 11 (0.0) | 608 (0.2) | 329557 |
| 80+ | 48169 (28.5) | 3167 (1.9) | 72485 (42.9) | 17596 (10.4) | 18403 (10.9) | 5416 (3.2) | 3319 (2.0) | 11 (0.0) | 295 (0.2) | 168861 |
| Race/ethnicity |  |  |  |  |  |  |  |  |  |  |
| Non-Hispanic white | 175195 (44.1) | 10197 (2.6) | 141813 (35.7) | 28286 (7.1) | 26886 (6.8) | 8294 (2.1) | 6045 (1.5) | 12 (0.0) | 653 (0.2) | 397381 |
| Non-Hispanic black | 38542 (41.9) | 2081 (2.3) | 34013 (37.0) | 6052 (6.6) | 7220 (7.9) | 2184 (2.4) | 1742 (1.9) | 4 (0.0) | 166 (0.2) | 92004 |
| Hispanic | 108152 (50.8) | 5485 (2.6) | 69505 (32.7) | 11801 (5.5) | 11544 (5.4) | 3405 (1.6) | 2633 (1.2) | 15 (0.0) | 353 (0.2) | 212893 |
| Non-Hispanic  Asian/pacific islander | 38774 (44.6) | 2063 (2.4) | 31043 (35.7) | 6196 (7.1) | 5641 (6.5) | 1750 (2.0) | 1310 (1.5) | 3 (0.0) | 198 (0.2) | 86978 |
| Non-Hispanic Native  American | 809 (48.9) | 38 (2.3) | 557 (33.7) | 94 (5.7) | 101 (6.1) | 26 (1.6) | 24 (1.5) | 0 (0.0) | 4 (0.2) | 1653 |
| Multiple | 847 (51.5) | 54 (3.3) | 487 (29.2) | 98 (6.0) | 95 (5.8) | 37 (2.3) | 30 (1.8) | 0 (0.0) | 4 (0.2) | 1646 |
| Other/Unknown | 5418 (56.7) | 260 (2.7) | 2826 (29.6) | 430 (4.5) | 360 (3.8) | 117 (1.2) | 122 (1.3) | 0 (0.0) | 15 (0.2) | 9548 |
| **Tricuspid stenosis** |  |  |  |  |  |  |  |  |  |  |
| Sex |  |  |  |  |  |  |  |  |  |  |
| Female | - | - | 118 (52.4) | 38 (16.9) | 24 (10.7) | 4 (1.8) | 11 (4.9) | 0 (0.0) | 30 (13.3) | 225 |
| Male | - | - | 78 (45.4) | 29 (16.9) | 21 (12.2) | 7 (4.1) | 13 (7.6) | 0 (0.0) | 24 (14.0) | 172 |
| Age group |  |  |  |  |  |  |  |  |  |  |
| 18-49 | - | - | 62 (47.3) | 50 (38.2) | 4 (3.1) | 1 (0.8) | 4 (3.1) | 0 (0.0) | 10 (7.6) | 131 |
| 50-64 | - | - | 35 (49.3) | 5 (7.0) | 9 (12.7) | 3 (4.2) | 11 (15.5) | 0 (0.0) | 8 (11.3) | 71 |
| 65-79 | - | - | 69 (55.7) | 5 (4.0) | 21 (16.9) | 4 (3.2) | 5 (4.0) | 0 (0.0) | 20 (16.1) | 124 |
| 80+ | - | - | 30 (42.3) | 7 (9.9) | 11 (15.5) | 3 (4.2) | 4 (5.6) | 0 (0.0) | 16 (22.5) | 71 |
| Race/ethnicity |  |  |  |  |  |  |  |  |  |  |
| Non-Hispanic white | - | - | 83 (49.1) | 33 (19.5) | 17 (10.1) | 6 (3.6) | 9 (5.3) | 0 (0.0) | 21 (12.4) | 169 |
| Non-Hispanic black | - | - | 24 (49.0) | 14 (28.6) | 4 (8.2) | 0 (0.0) | 4 (8.2) | 0 (0.0) | 3 (6.1) | 49 |
| Hispanic | - | - | 67 (49.3) | 20 (14.7) | 20 (14.7) | 4 (2.9) | 8 (5.9) | 0 (0.0) | 17 (12.5) | 136 |
| Non-Hispanic  Asian/pacific islander | - | - | 19 (50.0) | 0 (0.0) | 3 (7.9) | 1 (2.6) | 3 (7.9) | 0 (0.0) | 12 (31.6) | 38 |
| Non-Hispanic Native  American | - | - | 0 (0.0) | 0 (0.0) | 0 (0.0) | 0 (0.0) | 0 (0.0) | 0 (0.0) | 1 (100.0) | 1 |
| Multiple | - | - | 0 (0.0) | 0 (0.0) | 0 (0.0) | 0 (0.0) | 0 (0.0) | 0 (0.0) | 0 (0.0) | 0 |
| Other/Unknown | - | - | 3 (75.0) | 0 (0.0) | 1 (25.0) | 0 (0.0) | 0 (0.0) | 0 (0.0) | 0 (0.0) | 4 |
| **Tricuspid regurgitation** |  |  |  |  |  |  |  |  |  |  |
| Sex |  |  |  |  |  |  |  |  |  |  |
| Female | 210902 (45.8) | 11301 (2.5) | 159471 (34.7) | 26692 (5.8) | 33787 (7.3) | 9467 (2.1) | 8112 (1.8) | 19 (0.0) | 339 (0.1) | 460090 |
| Male | 228812 (51.6) | 10721 (2.4) | 150020 (33.8) | 20141 (4.5) | 23641 (5.3) | 5752 (1.3) | 4400 (1.0) | 9 (0.0) | 359 (0.1) | 621237 |
| Age group |  |  |  |  |  |  |  |  |  |  |
| 18-49 | 92627 (65.7) | 4466 (3.2) | 35576 (25.3) | 3246 (2.3) | 3218 (2.3) | 829 (0.6) | 807 (0.6) | 4 (0.0) | 130 (0.1) | 140903 |
| 50-64 | 123678 (57.7) | 5913 (2.8 | 65928 (30.8) | 7364 (3.4) | 7716 (3.6) | 1916 (0.9) | 1694 (0.8) | 4 (0.0) | 175 (0.1) | 214388 |
| 65-79 | 167337 (45.8) | 8545 (2.3) | 134515 (36.8) | 20333 (5.6) | 23740 (6.5) | 5915 (1.6) | 4840 (1.3) | 9 (0.0) | 286 (0.1) | 365520 |
| 80+ | 56083 (30.6) | 3098 (1.7) | 73479 (40.1) | 15891 (8.7) | 22755 (12.4) | 6559 (3.6) | 5171 (2.8) | 11 (0.0) | 107 (0.1) | 183154 |
| Race/ethnicity |  |  |  |  |  |  |  |  |  |  |
| Non-Hispanic white | 212581 (48.4) | 11162 (2.5) | 150831 (34.3) | 23819 (5.4) | 27795 (6.3) | 7405 (1.7) | 5429 (1.2) | 9 (0.0) | 314 (0.1) | 439345 |
| Non-Hispanic black | 44579 (41.8) | 2031 (1.9) | 39499 (37.1) | 6175 (5.8) | 9208 (8.6) | 2486 (2.3) | 2449 (2.3) | 0 (0.0) | 119 (0.1) | 106546 |
| Hispanic | 127913 (52.0) | 6209 (2.5) | 80737 (32.8) | 10890 (4.4) | 13464 (5.5) | 3485 (1.4) | 3156 (1.3) | 11 (0.0) | 186 (0.1) | 24051 |
| Non-Hispanic  Asian/pacific islander | 46213 (47.4) | 2225 (2.3) | 33997 (34.9) | 5464 (5.6) | 6430 (6.6) | 1692 (1.7) | 1354 (1.4) | 8 (0.0) | 71 (0.1) | 97454 |
| Non-Hispanic Native  American | 981 (53.7) | 37 (2.0) | 596 (32.6) | 73 (4.0) | 91 (5.0) | 24 (1.3) | 24 (1.3) | 0 (0.0) | 1 (0.1) | 1827 |
| Multiple | 1016 (53.9) | 67 (3.6) | 584 (31.0) | 74 (3.9) | 93 (4.9) | 20 (1.1) | 29 (1.5) | 0 (0.0) | 1 (0.1) | 1884 |
| Other/Unknown | 6442 (59.3) | 291 (2.7) | 3254 (30.0) | 339 (3.1) | 348 (3.2) | 107 (1.0) | 71 (0.7) | 0 (0.0) | 6 (0.1) | 10858 |
| **Pulmonic stenosis** |  |  |  |  |  |  |  |  |  |  |
| Sex |  |  |  |  |  |  |  |  |  |  |
| Female | - | - | 979 (70.0) | 84 (6.0) | 166 (11.9) | 24 (1.7) | 24 (1.7) | 4 (0.3) | 118 (8.4) | 1399 |
| Male | - | - | 779 (65.7) | 76 (6.4) | 134 (11.3) | 14 (1.2) | 42 (3.5) | 0 (0.0) | 140 (11.8) | 1185 |
| Age group |  |  |  |  |  |  |  |  |  |  |
| 18-49 | - | - | 1074 (67.5) | 108 (6.8) | 212 (13.3) | 33 (2.1) | 49 (3.1) | 4 (0.3) | 111 (7.0) | 1591 |
| 50-64 | - | - | 325 (69.0) | 31 (6.6) | 55 (11.7) | 3 (0.6) | 8 (1.7) | 0 (0.0) | 49 (10.4) | 471 |
| 65-79 | - | - | 258 (67.9) | 15 (4.0) | 26 (6.8) | 2 (0.5) | 2 (0.5) | 0 (0.0) | 77 (20.3) | 380 |
| 80+ | - | - | 101 (70.6) | 6 (4.2) | 8 (5.6) | 0 (0.0) | 7 (4.9) | 0 (0.0) | 21 (14.7) | 143 |
| Race/ethnicity |  |  |  |  |  |  |  |  |  |  |
| Non-Hispanic white | - | - | 680 (68.8) | 62 (6.3) | 92 (9.3) | 12 (1.2) | 25 (2.5) | 2 (0.2) | 115 (11.6) | 988 |
| Non-Hispanic black | - | - | 123 (63.7) | 11 (5.7) | 23 (11.9) | 6 (3.1) | 3 (1.6) | 0 (0.0) | 27 (14.0) | 192 |
| Hispanic | - | - | 770 (68.3) | 66 (5.9) | 147 (13.1) | 10 (0.9) | 33 (2.9) | 1 (0.1) | 95 (8.5) | 1122 |
| Non-Hispanic  Asian/pacific islander | - | - | 135 (64.9) | 18 (8.7) | 25 (12.0) | 9 (4.3) | 5 (2.4) | 1 (0.5) | 15 (7.2) | 208 |
| Non-Hispanic Native  American | - | - | 7 (100.0) | 0 (0.0) | 0 (0.0) | 0 (0.0) | 0 (0.0) | 0 (0.0) | 0 (0.0) | 7 |
| Multiple | - | - | 6 (50.0) | 2 (16.7) | 1 (8.3) | 1 (8.3) | 0 (0.0) | 0 (0.0) | 2 (16.7) | 12 |
| Other/Unknown | - | - | 37 (67.3) | 1 (1.8) | 13 (23.6) | 0 (0.0) | 0 (0.0) | 0 (0.0) | 4 (7.3) | 55 |
| **Pulmonic regurgitation** |  |  |  |  |  |  |  |  |  |  |
| Sex |  |  |  |  |  |  |  |  |  |  |
| Female | 97526 (69.9) | 2436 (1.8) | 33963 (24.3) | 2550 (1.8) | 2418 (1.7) | 231 (0.2) | 251 (0.2) | 0 (0.00) | 153 (0.1) | 139528 |
| Male | 101816 (69.1) | 2551 (1.7) | 37134 (25.2) | 2737 (1.9) | 2580 (1.8) | 211 (0.1) | 211 (0.1) | 0 (0.00) | 129 (0.1) | 147369 |
| Age group |  |  |  |  |  |  |  |  |  |  |
| 18-49 | 36628 (77.7) | 870 (1.8) | 7991 (16.9) | 583 (1.2) | 607 (1.3) | 127 (0.3) | 294 (0.6) | 0 (0.00) | 71 (0.2) | 47171 |
| 50-64 | 48999 (76.0) | 1105 (1.7) | 12745 (19.8) | 706 (1.1) | 699 (1.1) | 84 (0.1) | 71 (0.1) | 0 (0.00) | 67 (0.1) | 64476 |
| 65-79 | 78082 (68.2) | 2038 (1.8) | 30005 (26.2) | 2099 (1.8) | 1923 (1.7) | 127 (0.1) | 70 (0.1) | 0 (0.00) | 107 (0.1) | 114451 |
| 80+ | 35637 (58.6) | 974 (1.6) | 20356 (33.5) | 1899 (3.1) | 1769 (2.9) | 106 (0.2) | 27 (0.0) | 0 (0.00) | 37 (0.1) | 60850 |
| Race/ethnicity |  |  |  |  |  |  |  |  |  |  |
| Non-Hispanic white | 94375 (70.0) | 2519 (1.9) | 32871 (24.4) | 2365 (1.8) | 2139 (1.6) | 175 (0.1) | 184 (0.1) | 0 (0.00) | 110 (0.1) | 134738 |
| Non-Hispanic black | 22062 (63.4) | 541 (1.6) | 10338 (29.7) | 847 (2.4) | 885 (2.5) | 61 (0.2) | 51 (0.2) | 0 (0.00) | 38 (0.1) | 34823 |
| Hispanic | 55901 (72.3) | 1332 (1.7) | 17330 (22.4) | 1190 (2.4) | 1162 (1.5) | 146 (0.2) | 175 (0.2) | 0 (0.00) | 88 (0.1) | 77324 |
| Non-Hispanic  Asian/pacific islander | 23340 (66.4) | 514 (1.5) | 9567 (27.2) | 824 (2.3) | 767 (2.2) | 55 (0.2) | 46 (0.1) | 0 (0.00) | 40 (0.1) | 35153 |
| Non-Hispanic Native  American | 421 (70.6) | 15 (2.5) | 142 (23.8) | 10 (1.7) | 7 (1.2) | 0 (0.0) | 0 (0.0) | 0 (0.00) | 1 (0.2) | 2025 |
| Multiple | 470 (72.5) | 20 (3.1) | 134 (20.7) | 13 (2.0) | 9 (1.4) | 2 (0.3) | 0 (0.0) | 0 (0.00) | 0 (0.0) | 648 |
| Other/Unknown | 2777 (76.7) | 46 (1.3) | 715 (19.8) | 38 (1.1) | 29 (0.8) | 5 (0.1) | 6 (0.2) | 0 (0.00) | 5 (0.1) | 3621 |

TTE: Transthoracic echocardiogram

KPSC: Kaiser Permanente Southern California
