# Supplementary figures and images for: Identifying the Severity of Heart Valve Stenosis and Regurgitation Among a Diverse Population Within an Integrated Health Care System: Natural Language Processing Approach
Source: JMIR Cardio. 2024 Sep 30;8:e60503. doi: 10.2196/60503 (PMC11474122; doi:10.2196/60503)

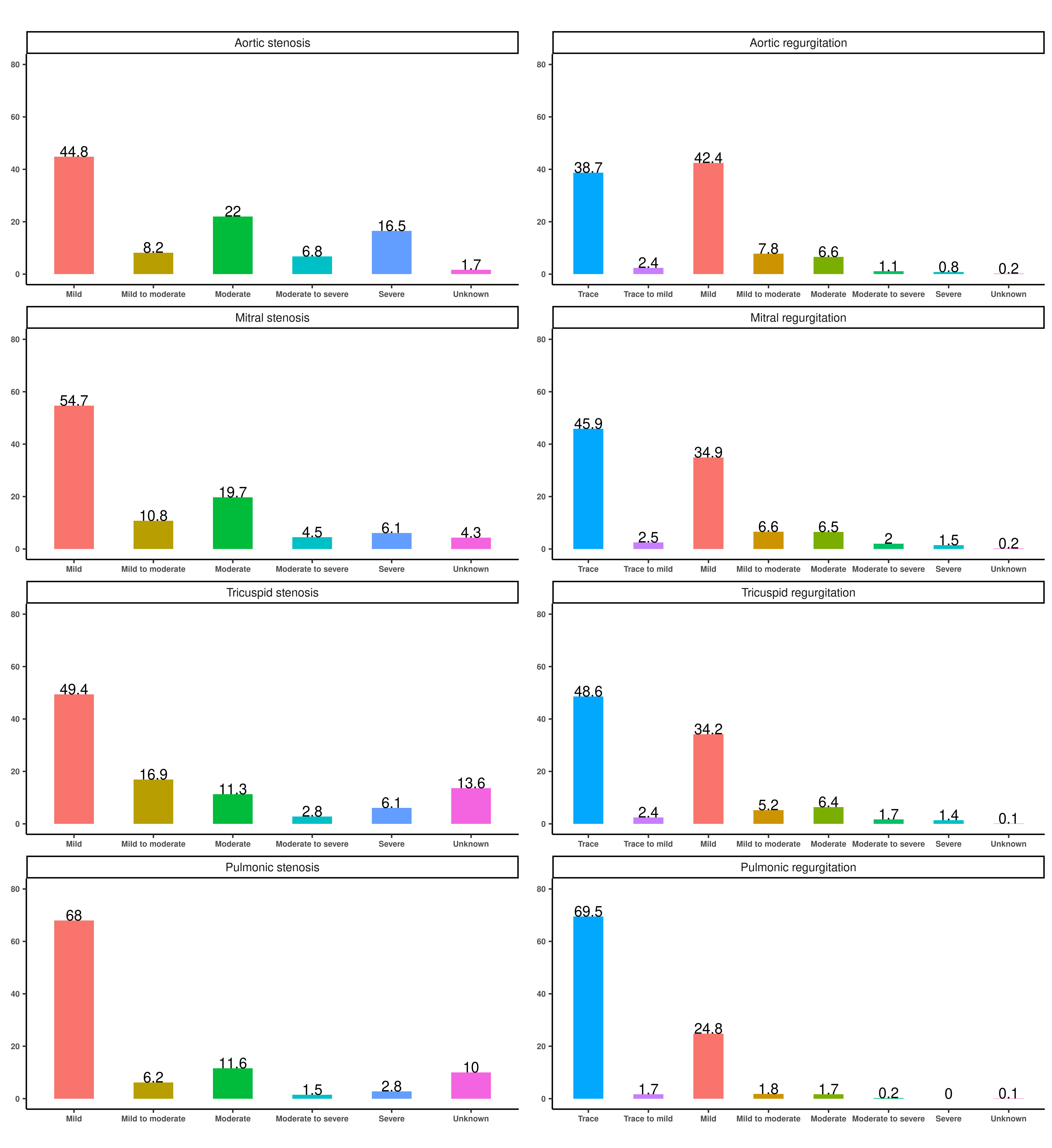

Supplement: Multimedia Appendix 2 [file cardio_v8i1e60503_app2.png]
